# Autophagy: a multifaceted player in the fate of sperm

**DOI:** 10.1093/humupd/dmab043

**Published:** 2021-12-30

**Authors:** Mei Wang, Ling Zeng, Ping Su, Ling Ma, Ming Zhang, Yuan zhen Zhang

**Affiliations:** 1 Center for Reproductive Medicine, Zhongnan Hospital of Wuhan University, Wuhan, Hubei, P.R. China; 2 Harvard Reproductive Endocrine Science Center and Reproductive Endocrine Unit, Department of Medicine, Massachusetts General Hospital, Harvard Medical School, Boston, MA, USA; 3 Clinical Medicine Research Center of Prenatal Diagnosis and Birth Health in Hubei Province, Wuhan, Hubei, P.R. China; 4 Institute of Reproductive Health, Tongji Medical College, Huazhong University of Science and Technology, Wuhan, Hubei, P.R. China

**Keywords:** autophagy / hypothalamic-pituitary-testis axis / spermatogenesis / acrosome biogenesis / acrosome reaction / paternal mitochondria elimination / ectoplasmic specialisation / sperm maturation / erectile dysfunction / fertilisation

## Abstract

**BACKGROUND:**

Autophagy is an intracellular catabolic process of degrading and recycling proteins and organelles to modulate various physiological and pathological events, including cell differentiation and development. Emerging data indicate that autophagy is closely associated with male reproduction, especially the biosynthetic and catabolic processes of sperm. Throughout the fate of sperm, a series of highly specialized cellular events occur, involving pre-testicular, testicular and post-testicular events. Nonetheless, the most fundamental question of whether autophagy plays a protective or harmful role in male reproduction, especially in sperm, remains unclear.

**OBJECTIVE AND RATIONALE:**

We summarize the functional roles of autophagy in the pre-testicular (hypothalamic–pituitary–testis (HPG) axis), testicular (spermatocytogenesis, spermatidogenesis, spermiogenesis, spermiation) and post-testicular (sperm maturation and fertilization) processes according to the timeline of sperm fate. Additionally, critical mechanisms of the action and clinical impacts of autophagy on sperm are identified, laying the foundation for the treatment of male infertility.

**SEARCH METHODS:**

In this narrative review, the PubMed database was used to search peer-reviewed publications for summarizing the functional roles of autophagy in the fate of sperm using the following terms: ‘autophagy’, ‘sperm’, ‘hypothalamic–pituitary–testis axis’, ‘spermatogenesis’, ‘spermatocytogenesis’, ‘spermatidogenesis’, ‘spermiogenesis’, ‘spermiation’, ‘sperm maturation’, ‘fertilization’, ‘capacitation’ and ‘acrosome’ in combination with autophagy-related proteins. We also performed a bibliographic search for the clinical impact of the autophagy process using the keywords of autophagy inhibitors such as ‘bafilomycin A1’, ‘chloroquine’, ‘hydroxychloroquine’, ‘3-Methyl Adenine (3-MA)’, ‘lucanthone’, ‘wortmannin’ and autophagy activators such as ‘rapamycin’, ‘perifosine’, ‘metformin’ in combination with ‘disease’, ‘treatment’, ‘therapy’, ‘male infertility’ and equivalent terms. In addition, reference lists of primary and review articles were reviewed for additional relevant publications. All relevant publications until August 2021 were critically evaluated and discussed on the basis of relevance, quality and timelines.

**OUTCOMES:**

(i) In pre-testicular processes, autophagy-related genes are involved in the regulation of the HPG axis; and (ii) in testicular processes, mTORC1, the main gate to autophagy, is crucial for spermatogonia stem cell (SCCs) proliferation, differentiation, meiotic progression, inactivation of sex chromosomes and spermiogenesis. During spermatidogenesis, autophagy maintains haploid round spermatid chromatoid body homeostasis for differentiation. During spermiogenesis, autophagy participates in acrosome biogenesis, flagella assembly, head shaping and the removal of cytoplasm from elongating spermatid. After spermatogenesis, through PDLIM1, autophagy orchestrates apical ectoplasmic specialization and basal ectoplasmic specialization to handle cytoskeleton assembly, governing spermatid movement and release during spermiation. In post-testicular processes, there is no direct evidence that autophagy participates in the process of capacitation. However, autophagy modulates the acrosome reaction, paternal mitochondria elimination and clearance of membranous organelles during fertilization.

**WIDER IMPLICATIONS:**

Deciphering the roles of autophagy in the entire fate of sperm will provide valuable insights into therapies for diseases, especially male infertility.

## Introduction

### Autophagy

**Figure 1.**
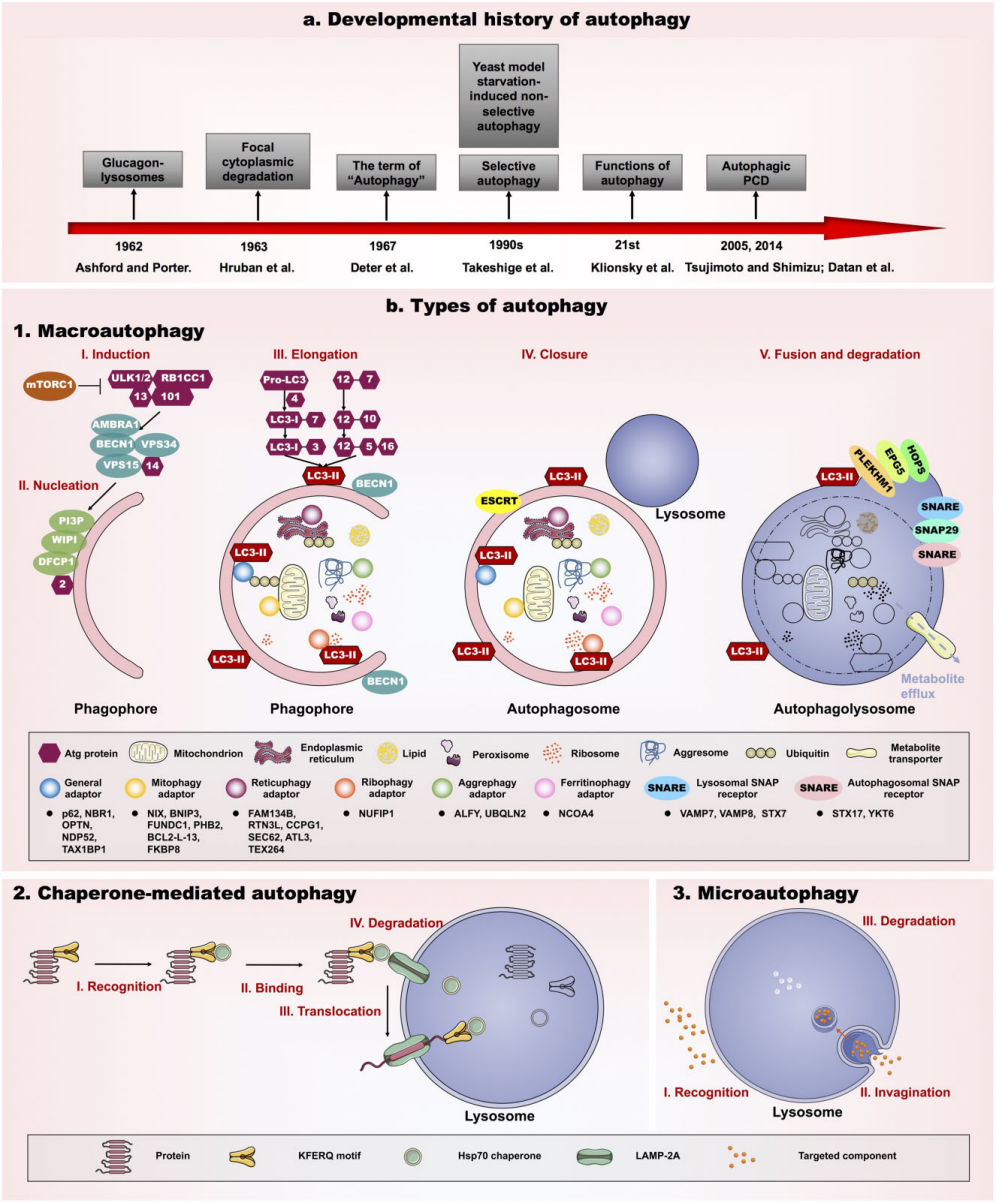
**The developmental history and types of autophagy.** (**a**) Developmental history of autophagy. In 1962, autophagy originated from an observation that increased lysosomes migrated toward organelles in response to the addition of glucagon in rat liver cells. In 1963, a detailed ultrastructure of ‘focal cytoplasmic degradation’ was described; the term ‘autophagy’ was proposed as a part of lysosomal function. In the 1990s, autophagy started to be investigated extensively due to starvation-induced non-selective autophagy, followed by selective autophagy. In the early 21st century, autophagy was demonstrated as one of the repair mechanisms as damaged organelles, membranes, and proteins were degraded for generating energy and building new proteins and membranes through autophagy-mediated cellular metabolism. In 2005 and 2014, autophagic cell death was identified as a novel way of programmed cell death (PCD). (**b**) Types of autophagy. 1. Macroautophagy is a conserved dynamic process, which consists of induction (phagophore formation), nucleation, elongation, closure (autophagosome formation), fusion (autophagosome and lysosome into autophagolysosome) and degradation, thus degrading organelles and proteins for the synthesis of new macromolecules or as a source of energy. Involved signaling pathways are as follows: I. Induction: mTORC1 inhibits ULK complex, which comprises ULK1/2 (also named ATG1), RB1CC1 (also named ATG17), ATG13 and ATG101, leading to the translocation of the complex to the phagophore and initiating the autophagy. II. Nucleation: ULK complex activates PI3K complex (BECN1-VPS34-VPS15-ATG14) by the phosphorylation of AMBRA1 and BECN1, generating phosphatidylinositol 3-phosphate (PI3P) on phagophore membrane. PI3P recruits WIPI, DFCP1 and ATG2 to promote autophagosome formation. III. Elongation: it is regulated by two conjugated systems of LC3-II (also named ATG8) and ATG12–ATG5–ATG16 complex. LC3 precursor is hydrolyzed by ATG4 to form LC3-I, which interacts with ATG7 and ATG3, forming LC3-II (also known as LC3-PE). IV. Closure: LC3-II and ESCRT regulate the closure of phagophore, thus facilitating the autophagosome formation. V. Fusion and degradation: after closure, the mature autophagosome and a lysosome fuse into an autophagolososome, which degrades the dispensable organelles and proteins. Autophagosomal SNAP receptors (STX17, YKT6) interact with SNAP29, which binding to lysosomal SNAP receptors (VAMP7, VAMP8 and STX7), together with HOPS complex, EPG5 and PLEKHM1 promoting the fusion. Due to the difference of destructive targets, macroautophagy is divided into non-selective autophagy targeting bulk cytosol and selective autophagy targeting specific cargos, such as mitochondrion, endoplasmic reticulum, lipid, peroxisome, ribosome, aggresome and ferritin. The specific cargo directly recognizes LC3 or indirectly recognizes LC3 through ubiquitin with specific cargo adapters, such as general adaptors (p62, NBR1, OPTN, NDP52, TAX1BP1), mitophagy adapters (NIX, BNIP3, FUNDC1, PHB2, BCL2-L-13, FKBP8), reticuphagy adapters (FAM134B, RTN3L, CCPG1, SEC62, ATL3, TEX264), ribophagy adapter (NUFIP1), aggrephagy adaptors (ALFY, UBQLN2), and ferritinophagy adaptor (NCOA4). 2. In chaperone-mediated autophagy (CMA), proteins carrying KFERQ motif are recognized by the Hsp70 chaperone, which interacts with lysosome membrane protein LAMP-2A, leading the translocation of the bound protein into the lysosome and degradation. 3. Microautophagy is characterized by the direct engulfment of cytoplasmic material into the lysosome through invagination and pinching off.

Autophagy is a ‘self-eating’ catabolic process of degrading cytoplasmic materials in lysosomes, which plays a fundamental role in various physiological or pathological processes (Mizushima and Levine, 2020). Our knowledge of autophagy is dramatically expanding day by day. The 60-year developmental history of autophagy (Fig.  1a) reflects the progress of science and leads to a new era in our understanding of autophagy in human health. Known as a double-edged sword, autophagy can serve as protective mechanism by eliminating damaged organelles and providing energy for cellular renovation, yet, autophagy may also contribute to cell damage (Shintani and Klionsky, 2004).

Autophagy-related genes (Atgs) and enzymes are identified in the three types of autophagy: macroautophagy, chaperone-mediated autophagy (CMA) and microautophagy (Fig.  1b). Firstly, macroautophagy, as the major form of autophagy, is a conserved dynamic process, which consists of induction (phagophore formation), nucleation, elongation, closure (autophagosome formation), fusion (autophagosome and lysosome into autophagolysosome) and degradation, thus degrading organelles and proteins for the synthesis of new macromolecules or as a source of energy. Due to the difference of destructive targets, macroautophagy (hereafter called autophagy) is divided into non-selective autophagy (targeting bulk cytosol) and selective autophagy (targeting specific organelles), such as mitophagy, lipophagy, pexophagy, ribophagy, reticulophagy, aggrephagy and ferritinophagy. The involved signaling pathways and key molecules are shown in Fig.  1b. Secondly, in CMA, proteins carrying the KFERQ motif are recognized by the Hsp70 chaperone, which interacts with lysosome membrane protein LAMP-2A, leading the translocation of the bound protein into the lysosome (Boya *et al.*, 2013). Thirdly, microautophagy is characterized by the direct engulfment of cytoplasmic material into the lysosome through invagination and pinching off. To sustain homeostasis, autophagy, together with the ubiquitin-proteasome system (UPS), constitutes the major cellular quality control systems for the degradation and disposal of organelles (Pohl and Dikic, 2019). Depending on these emerging regulatory processes and functions, autophagy regulates cell growth, survival and cell death (Dai *et al.*, 2020).

### The fate of sperm

As shown in Table  I, the knockdown/knockout (KD/KO) of Atgs in mammalian testis has shown the role of specific molecules in the regulation of distinct testicular cells. Sperm is the male reproductive cell or gamete that fuses with an oocyte to form a fertilized oocyte that develops into an individual, so sperm is the key to male fertility (Kimble and Page, 2007). The fate of sperm refers to an intricate process from the origin of sperm to its disappearance. Sperm is produced by spermatogenesis in the male reproductive gland, the testis, whose functions are controlled by the hypothalamic–pituitary–gonadal (HPG) axis. Once completing spermatogenesis in the testis, sperm move into the epididymis to mature into spermatozoa. Subsequently, mature sperm-containing semen is ejaculated into the female reproductive tract and for fertilization of the oocyte. Overall, the fate of sperm may be categorized as pre-testicular, testicular, post-testicular according to the timeline (Krausz, 2011).

**Table I:** Autophagy-related genes KD/KO in the mammalian testis/testicular cells.

Autophagy- related gene	Autophagy- related process	KD/KO model	Fertility	Phenotype	Functions in testis	Reference(s)
**Macroautophagy**						
***Atg5***	Phagophore formation	cKO in germ cells	Subfertile	Induce sperm counts and motility reduction, misshapen sperm heads and tails, abnormal mitochondria and acrosome distribution	Elongating spermatid development, sperm individualization during spermiogenesis	Huang *et al.* (2021)
		cKO in Sertoli cells	Infertile	Disrupt cytoskeleton structures and ectoplasmic specialization assembly	Ectoplasmic specialization assembly	Liu *et al.* (2016)
		cKO in Leydig cells	Subfertile	Suppress testosterone synthesis, affect sexual behavior	Testosterone synthesis	Gao *et al.* (2018)
***Atg7***	Phagophore, autophagosome formation	cKO in germ cells	Subfertile	Inhibit spermatozoa flagella biogenesis and cytoplasm removal	Spermatozoa flagella biogenesis and cytoplasm removal during spermiogenesis	Shang *et al.* (2016)
		cKO in germ cells	Infertile	A defect in acrosome biogenesis	Acrosome biogenesis	Wang *et al.* (2014)
		cKO in Sertoli cells	Subfertile	Disrupt cytoskeleton structures and ectoplasmic specialization assembly	Ectoplasmic specialization assembly	Liu *et al.* (2016)
		cKO in Leydig cells	Subfertile	Suppress testosterone synthesis, affect sexual behavior	Testosterone synthesis	Gao *et al.* (2018)
		KD in rat primary Leydig cells	–	Suppress testosterone biosynthesis	Testosterone biosynthesis	Ma *et al.* (2018)
		KD in rat primary Sertoli cells	–	Promote androgen-binding protein expression	Autophagic clearance of androgen-binding protein	Ma *et al.* (2015)
***Beclin 1***	Phagophore formation	KD in TM3 mouse Leydig cells	–	Decrease testosterone production	Steroidogenesis	Li *et al.* (2011)
***Tfeb***	Lysosomal biogenesis	KD in GC-1 mouse spg cells	–	Not affect spermatogonial differentiation, but significantly reduce cell migration in GC-1 cells	Spermatogonial cell migration	Liu *et al.* (2018)
***Atg9, Atg12, Atg14, Atg16L, LC3, Dram1, Lamp1, Lamp2, p62***	Phagophore, autophagosome, autolysosome formation	No KD/KO in testis				

**Chaperon-mediated autophagy**						
***Ppp1cc***	HSC70 substrate	KO mice	Infertile	Impair spermiogenesis, meiosis, induce polyploid spermatids	Spermiogenesis	Varmuza *et al.* (1999)
		KO mice	Infertile	Disruptions in spermatogenesis that begin during prepubertal testicular development, and continue into adulthood, often resulting in loss of germ cells to the point of Sertoli cell-only syndrome.	Chromatin condensation and acrosome development	Forgione *et al.* (2010)
		cKO in germ cells	Infertile	Induce sperm counts reduction, misshapen sperm	Spermiogenesis	Sinha *et al.* (2013)
***Lamp2a***	Chaperon-mediated autophagy	No KD/KO in testis				

**Mitophagy**						
***Atg32, Nix***	Mitophagy	No KD/KO in testis				

**mTORC1-autophagy pathway**						
***Mtor***	mTORC1/mTORC2 component	KD in rat primary Sertoli cells	–	Reduce androgen- binding protein expression	Autophagic clearance of androgen binding protein	Ma *et al.* (2015)
		cKO in Sertoli cells	Infertile	Induce testicular atrophy, loss of Sertoli cell polarity, germ cell premature release/apoptosis, loss of pachytene spermatocytes and spermatids, sperm abnormalities	Sertoli cell polarity, germ cell development through the pachytene spermatocyte stage	Boyer *et al.* (2016)
		cKO in germ cells	Infertile	Result in smaller testis and no sperm, impair spermatogonial proliferation	Spermatogonial proliferation and differentiation	Serra *et al.* (2017)
		cKO in germ cells	Subfertile	Induce age-dependent perturbation of testicular development, diminished spermatogonial pool and germ cell population	Spermatogonial proliferation and differentiation	Cao *et al.* (2020)
***Raptor***	mTORC1 component	cKO in germ cells	Infertile	Block spermatogonia proliferated and differentiation, result in Sertoli cell-only testes by adulthood	SSCs pool maintenance	Serra *et al.* (2019)
		cKO in germ cells	Infertile	Induce smaller testes and infertility	Meiotic arrest and sex chromosomes silence	Xiong *et al.* (2017)
		cKO in germ cells	Infertile	Impair spermatogenesis and induce progressive loss of spermatogonia	SSCs proliferation	Wang *et al.* (2017)
		cKO in Sertoli cells	Infertile	Cause severe tubular degeneration in the neonatal testis, azoospermia in adult mice with disruption of cytoskeletal organization	Sertoli cell cytoskeletal organization and polarity	Xiong *et al.* (2018)
***Rictor***	mTORC2 component	cKO in germ cells	Sterile	Impair spermatogonial differentiation potential, cell–cell junctions, BTB dynamics, and spermiogenesis	Spermatogonial differentiation and intercellular adhesion	Bai *et al.* (2018)
***Akt1/2***	Upstream of mTOR	KD in rat primary Sertoli cells	–	Disrupt Sertoli cell tight junction barrier	BTB function	Mok *et al.* (2015)
***Lama2***	Upstream of mTORC1	KD in rat primary Sertoli cells	–	Perturb F-actin and MTs organization in Sertoli cells	Sertoli cell BTB dynamics	Gao *et al.* (2017)
***Tsc1***	mTORC1 inhibitor	cKO in germ cells	Subfertile	Induce testicular developmental defects, partial spermatogenic arrest, excessive germ cell loss, sperm count reduction and subfertility; mTORC1 activation promotes spermatogonial differentiation at the expense of germline maintenance	Spermatogonial differentiation	Wang *et al.* (2016)
***Bif1, Uvrag, Ambra1***	Downstream of mTORC1	No KD/KO in testis				
***ULK1***	ULK complex	KD in goat primary Sertoli cells	–	Decrease cell viability and expressions of goat Sertoli cell marker genes (*ABP*, *AMH*, *FASL* and *GATA4*)	Sertoli cell function, viability	Pang *et al.* (2019)
***ULK2***	ULK complex	KD in swine Sertoli cells	–	Inhibit swine Sertoli cell autophagy	Sertoli cell function	Ran *et al.* (2018)
***Atg13, Atg101, Fip200***	ULK complex	No KD/KO in testis				
***Vps34, Vps15, Atg14L***	VPS34 complex, autophagosome formation	No KD/KO in testis				

*ABP*, sex hormone binding globulin; *AMH*, anti-Mullerian hormone; BTB, blood-testis barrier; cKO, conditional knockout; *FASL*, Fas ligand; *GATA4*, GATA binding protein 4; GC-1 cells, mouse spermatogonial cell lines; KD, knockdown; KO, knockout; MT, microtubule; mTOR, mammalian target of rapamycin; SCC, spermatogonia stem cell; TM3 cells, mouse Leydig cell lines; ULK1, Unc-51 like autophagy activating kinase.

In pre-testicular processes, the HPG axis is the neuroendocrine network that regulates sexual development and reproduction (Navarro and Tena-Sempere, 2011). In the hypothalamus, kisspeptin (a neuropeptide), following neurokinin B and dynorphin, signals directly to GnRH neurons to orchestrate pulsatile GnRH release (Skorupskaite *et al.*, 2014). GnRH binds to a membrane receptor in the pituitary, which secretes LH and follicle-stimulating hormone (FSH) (Kaprara and Huhtaniemi, 2018). After release from the pituitary, LH and FSH interact with the LH/FSH receptor in testicular cells, respectively, to initiate and maintain spermatogenesis.

**Figure 2.**
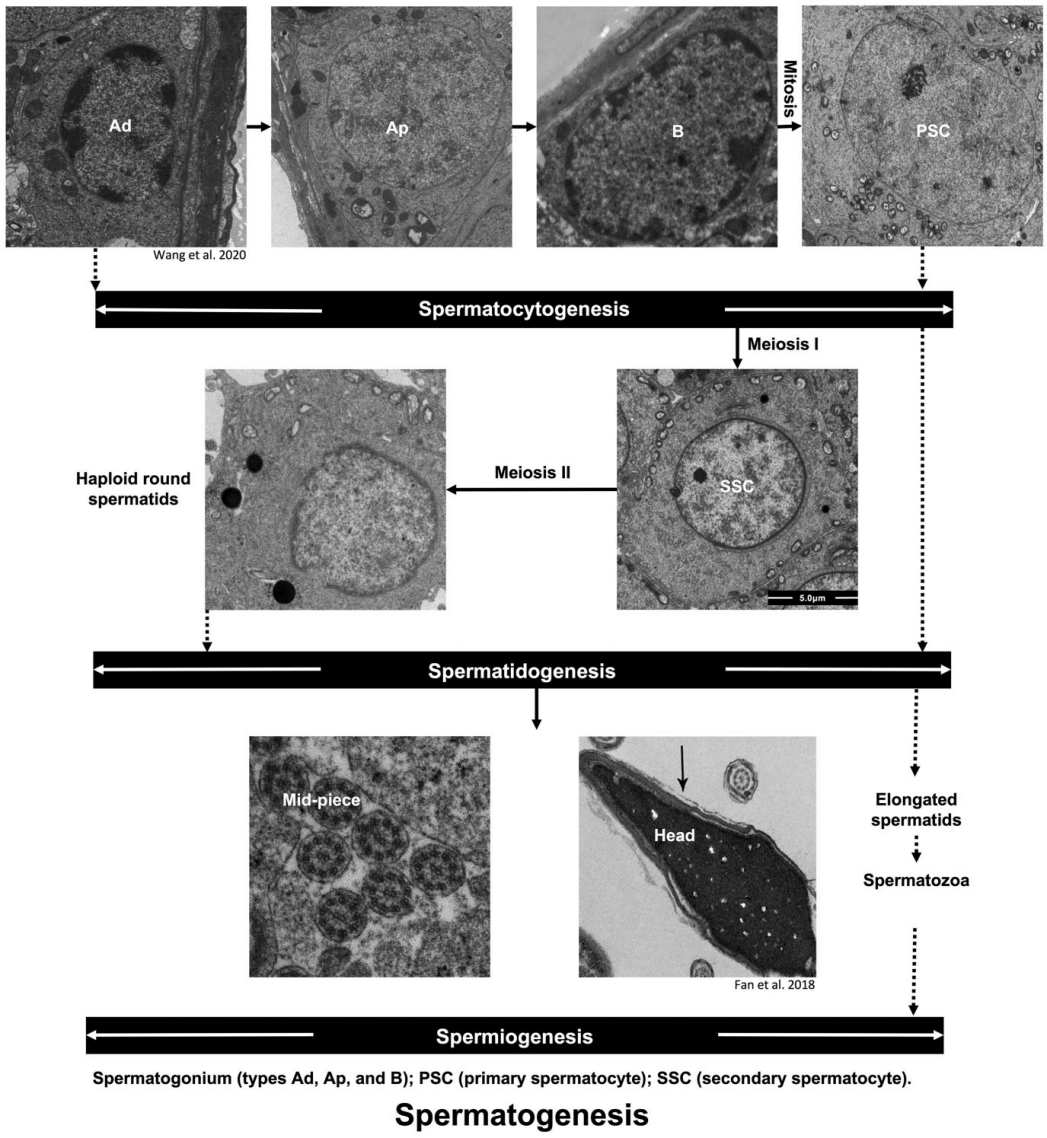
**The process of spermatogenesis.** The entire process of spermatogenesis can be divided into three stages as follows: Spermatocytogenesis: spermatogonia undergo mitosis to develop into primary spermatocytes; Spermatidogenesis: the primary spermatocytes undergo meiosis I to form secondary spermatocytes, which divide into haploid round spermatids via meiosis II; and spermiogenesis: round spermatids are differentiated into the elongated spermatids and then spermatozoa.

In testicular processes, the testis is responsible for producing spermatozoa through spermatogenesis. Testicular deficiency often leads to spermatogenic failure caused by conditions other than obstruction or HPG dysfunction. The entire process of spermatogenesis can be divided into three stages (Fig.  2), as follows: (i) during spermatocytogenesis, spermatogonia undergo mitosis to develop into primary spermatocytes (PSC); (ii) during spermatidogenesis, the PSC undergo meiosis I to form secondary spermatocytes, which divide into haploid round spermatids via meiosis II; and (iii) during spermiogenesis, round spermatids are differentiated into the elongated spermatids and then spermatozoa (Cheng and Mruk, 2012; Staub and Johnson, 2018). The last step in the testis is spermiation. Elongated spermatids are then released from the Sertoli cells into the seminiferous tubule lumen.

In post-testicular processes, after spermatogenesis and spermiation, spermatozoa are transported from the testis to the epididymis for obtaining progressive motility and fertility (Sullivan and Mieusset, 2016). Subsequently, mature sperm are ejaculated into the female reproductive tract to undergo capacitation and activation (Jin and Yang, 2017). Eventually, the fusion of the sperm and the oocyte into a zygote, namely fertilization, represents the disappearance of sperm and appearance of a new individual, which indicates the final step for the fate of sperm.

Recently, increasing evidence has shown that autophagy functions in multiple mammalian organs or systems, including the embryo (Yoshii *et al.*, 2016), placenta (Agrawal *et al.*, 2015), liver (Komatsu *et al.*, 2005), brain (Kaushik *et al.*, 2011), heart (Nakai *et al.*, 2007; Lu *et al.*, 2016), skeletal muscle (Masiero *et al.*, 2009), intestine (Cadwell *et al.*, 2008), pancreas (Ebato *et al.*, 2008), kidney (Hartleben *et al.*, 2010), and the male (Gao *et al.*, 2018) and female reproductive systems (Gawriluk *et al.*, 2014) (Table  II). Atgs have been implicated in numerous cellular events in the fate of sperm, such as GnRH secretion, LH, FSH and testosterone production, ectoplasmic specialization (ES) assembly, acrosome biogenesis and paternal mitochondria elimination (PME), shedding light on new therapeutic targets in the autophagy pathway for male subfertility or infertility (Nian *et al.*, 2019). To date, there has been no effective and timely summary on the role of autophagy in the entire fate of sperm. Given that autophagy is known as a double-edged sword, the most fundamental question of whether autophagy plays a protective or a harmful role in the regulation of sperm remains unclear. In this narrative review, we will summarize the role of autophagy in the entire fate of sperm, including pre-testicular (HPG axis), testicular (spermatogenesis and spermiation) and post-testicular (sperm maturation and fertilization), and evaluate the clinical impact of autophagy on the sperm and male fertility. Our review will provide a better understanding of autophagy in the fate of sperm, laying the foundation for the further study of sperm, which can help us identify more therapeutic targets for male subfertility or infertility.

**Table II:** The functions of autophagy in mammalian systems.

Organs	Functions	Autophagy-related gene or protein changes	Selected references
**The male reproductive system**	Testosterone biosynthesis	Atg5/7↓	Gao *et al.* (2018) and Ma *et al.* (2018)
	Acrosome biogenesis	Atg7, Sirt1, Tbc1d20↓	Liu *et al.* (2017), Sidjanin *et al.* (2016) and Wang *et al.* (2014)
	Spermiogenesis	Atg7↓	Shang *et al.* (2016)
	Ectoplasmic specialization	Atg5/7↓	Liu *et al.* (2016)
	Androgen binding protein metabolism	Atg7, mTOR↓	Ma *et al.* (2015)
**The female reproductive system**	Preventing excessive loss of oocytes in the neonatal ovaries	Beclin 1, Atg7↓	Gawriluk *et al.* (2011) and Song *et al.* (2015)
	Promoting progesterone synthesis	Beclin 1↓	Gawriluk *et al.* (2014)
	Follicle atresia	LC3-II/LC3-I↑	Choi *et al.* (2011) and Serke *et al.* (2009)
**Embryogenesis**	Preimplantation development/protein synthesis	Atg5, Beclin 1↓	Tsukamoto *et al.* (2008) and Yue *et al.* (2003)
	Nervous system development	Ambra1↓	Fimia *et al.* (2007)
	Preventing neonatal lethality/survival during neonatal starvation	Atg3/5/7/8/9a/16L1↓	Komatsu *et al.* (2005), Kuma *et al.* (2004), Saitoh *et al.* (2008, 2009), Sou *et al.* (2008) and Yoshii *et al.* (2016)
**Placenta**	Preventing placental infection and preterm labor	LC3-II/LC3-I, Atg4/7/16L1↑ and p62↓	Agrawal *et al.* (2015) and Cao *et al.* (2016)
**Liver**	Constitutive turnover of cytoplasmic components	Atg7↓	Komatsu *et al.* (2005)
**Brain**	Regulating food intake and energy balance	Atg7↓	Kaushik *et al.* (2011)
	Metabolic regulation/central control of feeding, energy and body weight balance	Atg5/7, LC3-II/LC3-I↓	Meng and Cai (2011)
	Prevent neurodegenerative disease	Atg5/7, LC3-II/LC3-I, Atg12↓	Hara *et al.* (2006) and Komatsu *et al.* (2006)
	Axonal homeostasis	Atg5/7↓	Komatsu *et al.* (2007) and Nishiyama *et al.* (2007)
		Atg9, ULK1, Beclin 1, LC3-II↓	Ko *et al.* (2020)
**Heart**	Maintaining cardiomyocyte size and global cardiac structure and function/adaption to hemodynamic stress	Atg5/7, LC3-II/LC3-I↓ and p62↑	Nakai *et al.* (2007)
	Angiogenesis	Beclin 1, Atg5, LC3-II/LC3-I ↓ and p62↑	Lu *et al.* (2016)
**Skeletal Muscle**	Preserving muscle mass and to maintain myofiber integrity	Atg7↓	Masiero *et al.* (2009)
**Intestine**	Maintaining Paneth cells function	Atg5/16L1↓	Cadwell *et al.* (2008)
**Pancreas**	Maintaining pancreatic β-cell volume and function	Atg7↓	Ebato *et al.* (2008) and Jung *et al.* (2008)
	Adaption to high-fat diet	Atg7↓	Ebato *et al.* (2008)
**Kidney**	Maintaining podocyte integrity	Atg5↓	Hartleben *et al.* (2010)
	Maintaining proximal tubule cell homeostasis and protecting against ischemic injury	Atg5↓	Kimura *et al.* (2011)

mTOR, mammalian target of rapamycin.

## Pre-testicular processes

### Role of autophagy in HPG axis

*Atg5* deficient mice show neuronal dysfunction and hypogonadism (Yoshii *et al.*, 2016), implying the key role of autophagy in HPG axis. Warburg Micro syndrome (WARBM) is a rare autosomal recessive genetic disease characterized by defective neurodevelopmental and ophthalmological phenotypes, such as microcephaly, microcornea, optic atrophy, lower limb spasticity and hypogonadotropic hypogonadism (Nian *et al.*, 2019). *RAB18*, a member of small G protein, has been demonstrated to be a causative gene of WARBM in human. *Rab18*−/− mice neurons exhibit abnormal lysosomal transport and autophagosome marker LC3-II expression, suggesting the involvement of aberrant autophagy activities. Furthermore, RAB18 protein colocalizes with the lysosomal regulator RAB7 protein, which is upregulated in *Rab18*-deficient neurons, indicating a compensatory effect of *Rab7* (Nian *et al.*, 2019).

*Rab3GAP2*, another causative gene of WARBM, is reported to be a guanosine nucleotide exchange factor for activating *Rab18*. *Rab3GAP2* mutation in *Drosophila* leads to the disorganization of autophagosomals/late endosomal compartments and lysosomal transport, finally perturbing autolysosome morphology. Meanwhile, Rab3GAP2, RAB18 and ATG6/BECN1 (subunits of Vps34 complexes) are co-located on autophagosomal and autolysosomal membranes. Consequently, the Rab3GAP-RAB18 module regulates autolysosomal maturation via its interaction with the Vps34 complexes. Taken together, Rab3GAP-RAB18-VPS34-mediated autolysosomal maturation may contribute to the development of WARBM, affecting gonadal function (Takats *et al.*, 2021).

In addition to *Rab3GA*P and *RAB18*, mutations in GnRHR are also associated with hypogonadotropic hypogonadism. Houck *et al.* (2014) show that a missense mutation E90K in *GnRHR* induces misfolded proteins due to molecular chaperones Hsp70 and Hsp40 JB12. Interaction between the Hsp40 and Vps34 (autophagy initiation) complex permits the selective degradation of membrane proteins via an autophagy pathway.

Microcystin-leucine arginine (MC-LR), a kind of toxin produced by cyanobacterial, is reported to induce GnRH neurons apoptosis, resulting in a reduction of serum testosterone and spermatogenesis disruption in mice. An autophagy inhibitor (3-MA, PI3K inhibitor) aggravates MC-LR-induced apoptotic cell death in GT1-7 mouse hypothalamic GnRH neuronal cell line, implying a potential protective role of PI3K from apoptosis of GnRH neurons upon MC-LR exposure (Jin *et al.*, 2021).

GnRH and kisspeptin/neurokinin B/dynorphin neurons in the hypothalamus regulate the age-related physiological decline in energy metabolism, hormone regulation and reproduction. Mammalian target of rapamycin (mTOR), autophagy and SIRT1 have been recognized as critical factors or pathways in hypothalamus-mediated aging progression (Kim and Choe, 2019).

*ZNF216* is an identified causative gene for Gordon Holmes syndrome, characterized by ataxia, dementia and hypogonadotropic hypogonadism in humans. KD of *RNF216* leads to the augmentation of BECN1 and migration defects in the GN11 immature GnRH neuronal cell line, which can be rescued by the autophagy inhibitors chloroquine (CQ) and 3-MA. Meanwhile, rapamycin (an autophagy activator) can suppress the GN11 cell migration. Therefore, RNF216 regulates GnRH neuron migration via inhibiting BECN1-mediated autophagy, suggesting a potential contribution of autophagy to hypogonadotropic hypogonadism (Li *et al.*, 2019).

*KiSS1* plays a key role in the activation of the HPG axis in regulating the onset of puberty and reproductive function. *KiSS1*-derived peptides, i.e. kisspeptins, signal through the G-protein coupled receptor GPR54 (Corno and Perego, 2019). In breast cancer cells, *KiSS1* has been reported to down-regulate two Atgs (*ATG5* and *ATG7*) and inhibit conversion of LC3-I to LC3-II, whereas *KiSS1* KD leads to the down-regulation of p62 and up-regulation of Beclin 1. Therefore, *KiSS1* inhibition is associated with promotion of autophagy (Kaverina *et al.*, 2017).

Kisspeptin preserves mitochondrial function by inducing mitophagy and autophagy in the hippocampus of aging rat brains and in a human neuronal cell line via a series of signaling pathways, including Ca2+/CaM-dependent protein kinase β (CaMKKβ), AMP-activated protein kinase (AMPK) and Unc-51 like autophagy activating kinase (ULK1) (Mattam *et al.*, 2021).

Environmental endocrine disruptors can disturb HPG axis by autophagy. Cadmium (Cd) decreases the serum concentrations of GnRH, FSH, LH and testosterone. Naringenin (Nar) suppresses MDA and H_2_O_2_ production and protects the testis from Cd-induced autophagy by downregulating P62 and LC3-II expression. Therefore, Nar protects the testis from Cd-induced toxicity (Wang *et al.*, 2021). Paternal exposure to arsenic results in oxidative stress, autophagy and mitochondrial impairment in the HPG axis of pubertal male offspring. Specifically, autophagic cell death-related genes and proteins, such as *Atg3*, *Atg5*, *Beclin 1*, *p62*, *Atg12*, PI3K and mTOR, are disturbed in the HPG tissues of the pubertal male mice offspring (F1-generation) (Ommati *et al.*, 2019). Similarly, in the mature male offspring, arsenic induces autophagic alterations and mitochondrial impairments in the HPG-sperm axis through AMPK/tuberous sclerosis complex (TSC) (tuberous sclerosis complex)/mTOR and LC3-related pathways (Ommati *et al.*, 2020). Zearalenone, a non-steroidal estrogen mycotoxin, can induce mitochondrial dysfunctions through overproduction of reactive oxygen species (ROS) and aberrant autophagy pathways, finally disturbing the synthesis and secretion of mammalian sex steroid hormones (Zheng *et al.*, 2019).

Autosomal dominant familial neurohypophyseal diabetes insipidus (adFNDI) is a progressive and inherited neurodegenerative disorder. The mutation of vasopressin (VP) from posterior pituitary nerve terminals can cause adFNDI, which is rescued by the inhibition of autophagy. Consequently, autophagy is a prosurvival mechanism in cells expressing an adFNDI mutant VP transgene (Castino *et al.*, 2005a). Subsequently, Castino *et al.* (2005b) demonstrated that autophagy-mediated cell death is a two-hit process: the first hit of autophagy by cellular stress degrades the misfolded mutant protein, thus autophagy is pro-survival; whereas, a second insult triggers an autophagy-dependent apoptosis.

## Testicular processes

Spermatogenesis is a dynamic process that allows the development of a diploid spermatogonium (SG) into haploid elongated spermatids in the seminiferous tubules (Wang and Proud, 2011). In this process, a well-defined progression of mitosis, meiosis and morphological transformations occurs in spermatogonia, spermatocytes and spermatids (Griswold, 2016). Normal spermatogenesis requires a balance of degradation and energy supply to maintain cellular metabolic homeostasis. Interestingly, autophagy is a unique catabolic pathway that participates in diverse physiological processes, especially cell residual bodies disposal, structural reconstruction, growth and development (Dikic and Elazar, 2018). Increasing studies have revealed that autophagy is involved in multifarious environmental toxicant-induced injury of testicular cells, including Sertoli cells, Leydig cells, spermotogonium and primary and second spermatocytes (Table  III). Deciphering the role of autophagy in spermatogenesis will provide insights into the treatment of male infertility. Herein, we will clarify the role of autophagy in spermatogenesis (spermatocytogenesis, spermatidogenesis and spermiogenesis) (Roosen-Runge, 1962; Hess and Renato de Franca, 2008) and spermiation.

**Table III:** Autophagy in environmental toxicants-induced mammalian testicular cell injury.

Toxin (environmental source)	Affected testicular cell	Autophagy- related gene or protein changes	Resulted in testicular pathology	In human/animal/ cell model	Reference
**DEHP**	Leydig cells	LC3-II, Atg5, Beclin 1↑	Decrease serum testosterone, induce oxidative stress and cell apoptosis	Kunming mice	Sun *et al.* (2018)
	GC-1 cells	LC3-II, Beclin 1, Atg5, LC3-II/ LC3-I↑	Induce oxidative stress, increase autophagic vacuoles number	Mouse GC-1 spg cell line	Gan *et al.* (2020)
**Bisphenol A and nonylphenol**	Leydig cells and sperm	Beclin 1, Atg5/12, LC3↑	Induce spermatogenic epithelium atrophy, germ cell loss, changes of hormones in serum and oxidative stress	Prepubertal Sprague Dawley rats	Su *et al.* (2018)
**Copper**	GC-1 cells	Atg3, Atg5, p62, LC3-II/LC3-I, Beclin 1, Atg5, p62↑	Alter cell viability and morphology, induce oxidative stress-mediated mitochondrial dysfunction	Mouse GC-1 spg cell line	Kang *et al.* (2019)
**Aflatoxin B1**	Leydig cells and sperm	LC3, Beclin 1, Atg5, p62↑ p-mTOR/mTOR↓	Reduce serum testosterone level, impair sperm, induce the atrophic seminiferous tubules, vacuole-like changes of spermatogenic epithelium, and oxidative stress	Kunming mice	Huang *et al.* (2019)
	Leydig cells	LC3, Beclin 1, p62↑	lower serum T, LH and FSH levels, reduce Leydig cell number	Sprague Dawley rats	Chen *et al.* (2019b)
**Cisplatin **	Leydig cells	LC3-II, Atg5↑	Inhibit cell proliferation and vitality	MLTC-1 cell line	Yang *et al.* (2018)
**TOCP**	Leydig TM3 cells	LC3-II/LC3-I, Atg5, Beclin 1 ↑	Inhibit cell viability and testosterone output, increase autophagic vacuoles and oxidative stress	Mouse Leydig TM3 cell line	Liu *et al.* (2016)
	SSCs	LC3-II/LC3-I, Atg5, Beclin 1 ↑	Inhibit viability and proliferation of rat SSCs	Primary SSCs of rats	Liu *et al.* (2015)
**Fluoride**	Sertoli cells	Beclin 1, p62↑ LC3, Atg5↓	No significant morphological alterations	Primary Sertoli cells of mice	Feng *et al.* (2019)
	Leydig cells	LC3, Beclin 1, Atg5↑	Increase autophagosomes number	Kunming mice	Zhang *et al.* (2017)
**4-Nonylphenol**	Sertoli cells	Beclin 1, Atg3/5/7/12↑ LC3-II/LC3-I↑	Stimulate the formation of autophagosomes	Sprague Dawley rats	Duan *et al.* (2017)
**SCOTP**	SSCs	LC3-II/LC3-I, Atg5, Beclin 1↑	Decrease cell viability and increase autophagic vacuoles	Primary SSCs of rats	Xu *et al.* (2016)
**Arsenic**	Leydig tumor cells	LC3, Atg7, Beclin 1, Vps34↑	Impair lysosomes function and induce accumulation of autophagosomes	MLTC-1	Liang *et al.* (2020)
**Zinc oxide nanoparticles**	Leydig cells	LC3-II, Atg5, Beclin 1↑	Disrupt seminiferous epithelium, decrease sperm density and serum testosterone levels	Kunming mice and TM3 cells	Shen *et al.* (2019)
**Methyl mercury**	Sperm	LC3-II, Beclin 1↑ p-mTOR/mTOR↓	Reduce sperm count and motility, impair the seminiferous tubule, induce apoptosis and oxidative stress	Sprague Dawley rats	Chen *et al.* (2019a)
**Nicotine**	Leydig cells	Beclin 1, LC3B↑	Induce serum testosterone reduction and more autophagosomes	C57BL/6 J mice and TM3 cells	Zhao *et al.* (2018)
**Cadmium**	SSCs	Beclin 1, LC3B↑	Increase testicular organ coefficients, seminiferous tubular atrophy, SSCs falling off the inner lining, reduce germ cell layers of disorderly arrangements	Wistar rats	Wang *et al.* (2017)
	Spermotogonium, Sertoli/ Leydig cells, primary/second spermatocytes, GC-1/GC-2/TM3/TM4 cells	Beclin 1, LC3-II/LC3-I↑ p-mTOR/mTOR (*in vivo*)↓	Reduce sperm count and motility, impair the seminiferous tubule, induce autophagy, apoptosis and oxidative stress	Sprague Dawley rats, GC-1/GC-2/TM3/TM4 cells	Wang *et al.* (2020)
**Acrolein**	Leydig cells	Beclin 1↑	Suppress proliferation and viability of Leydig cells, decrease testosterone, stimulate autophagy	Primary Leydig cells of mice	Gu *et al.* (2017)

DEHP, di-2-ethyl hexyl phthalate;  GC-1 cells, mouse spermatogonial cell lines; GC-2 cells, mouse spermatocyte cell lines; MLTC-1, mouse Leydig tumor cell lines; mTOR, mammalian target of rapamycin; SCOTP, saligenin cyclic-*O*-tolyl phosphate; SSCs, spermatogonial stem cells; TM3 cells, mouse Leydig cell lines; TM4 cells, mouse Sertoli cell lines; TOCP, tri-ortho-cresyl phosphate.

### Role of autophagy in spermatocytogenesis

Spermatocytogenesis is the first stage of spermatogenesis. In this process, a diploid spermatogonial stem cell (SSC) undergoes mitosis to renew the stem cell pool or differentiate into two diploid PSC (Staub and Johnson, 2018). In non-primates, spermatogonia are composed of A-single (As), A-paired (Apr) and A-aligned (Aal), and Type B spermatogonia (Lie *et al.*, 2009). In primates, this compartment consists of three subtypes (Fig.  2): (i) Type A dark (Ad), namely SSCs, renew themselves or generate differentiating Type A pale (Ap) spermatogonia; (ii) Type A pale (Ap) undergo mitosis to acquire Type B spermatogonia; and (iii) Type B spermatogonia produce PSC (Clermont, 1966; Di Persio *et al.*, 2017). In terms of the causal relationship of Ap and Ad spermatogonia, other seemingly contradictory views are also proposed, such as that Ap spermatogonia are the SSCs, or that both of Ap and Ad cells are the SSCs simultaneously (Fouquet and Dadoune, 1986; Hermann *et al.*, 2009). Regardless of the debate on Ap and Ad, what can be confirmed is that Type B spermatogonia will differentiate into PSC via mitosis. In this process, autophagy delicately exerts its bilateral effects.

#### Mammalian target of rapamycin

mTOR, a serine/threonine kinase, is a major regulator of cell growth, survival, metabolism and immunity (Saxton and Sabatini, 2017). As a core component, mTOR forms two distinct signaling complexes, mTOR Complex 1 (mTORC1) and mTORC2 by binding specific proteins Raptor and Rictor, respectively (Kim *et al.*, 2002; Sarbassov *et al.*, 2004; Jacinto *et al.*, 2006). Differences in Raptor and Rictor sensitivity to rapamycin determine the differences in mTORC1 and mTORC2 function (Jacinto *et al.*, 2004; Sarbassov *et al.*, 2006). mTORC2 can phosphorylate Akt at Ser^473^ (Sarbassov *et al.*, 2005) and regulate cell survival, metabolism and cytoskeletal organization (Cybulski and Hall, 2009) via AGC family kinases (PKA, PKG and PKC) (Cybulski *et al.*, 2009). mTORC1 mainly regulates cell growth, proliferation, apoptosis, energy metabolism and autophagy (Kim *et al.*, 2002; Yang *et al.*, 2013). Noticeably, mTORC1 is the main gateway to autophagy (Rabanal-Ruiz *et al.*, 2017) by modulating the localization of transcription factor EB, a major transcriptional regulator of lysosomal and autophagy genes (Settembre *et al.*, 2013; Kim and Guan, 2015). Activation of mTORC1 by various nutrients and growth factors leads to the inhibition of autophagy through the phosphorylation of multiple autophagy-related proteins, such as ULK1, ATG13, AMBRA1 and ATG14L, which normally promote autophagy initiation and autophagosome nucleation (Kim and Guan, 2015).

Increasing studies have shown that mTOR regulates sperm quality in human (Silva *et al.*, 2015, 2019). Diverse environmental toxicants, such as cadmium, di-2-ethyl hexyl phthalate, fine particle matters, nonylphenol and silica nanoparticles, induce testicular injury and regulate autophagy via mTOR signaling in mice and rats (Huang *et al.*, 2016; Li *et al.*, 2017; Ren *et al.*, 2019, 2020; Zhang *et al.*, 2020b). In mammals, sperm count, motility and morphology present a significant positive correlation with the phosphorylated levels of p70S6 kinase (Silva *et al.*, 2015; Xu *et al.*, 2016). Rapamycin inhibits spermatogenesis by suppressing mTOR-p70S6 kinase to alter the autophagy status in male rats (Liu *et al.*, 2017b). Herein, this section will review the role of mTORC1 in spermatogenesis to indicate the regulation of sperm fate by autophagy.

**Figure 3.**
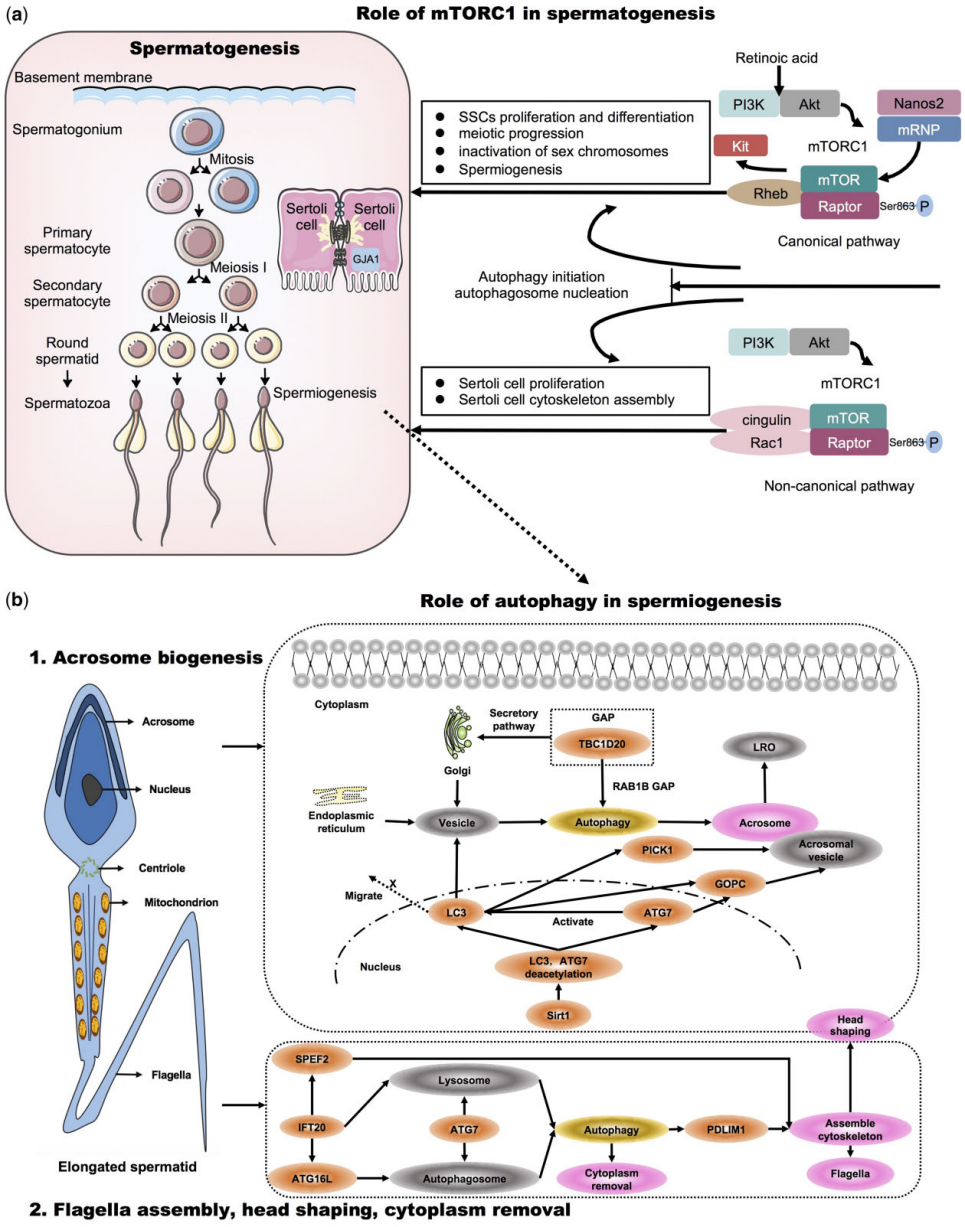
**Role of autophagy in spermatogenesis.** (**a**) Role of mTORC1 in spermatogenesis. mTORC1, the main gate to autophagy, is crucial for spermatogonia stem cell (SCCs) proliferation, differentiation, meiotic progression, inactivation of sex chromosomes and spermiogenesis. Firstly, proliferation of SSCs requires phosphorylation of the mTORC1 component Raptor at Ser^863^. Secondly, conditional ablation of *Raptor* causes infertility due to meiotic arrest and impaired inactivation of sex chromosomes in the male germline. Thirdly, *Rheb*, a critical component for mTORC1 activation, is required for meiotic progression and spermiogenesis. Consistently, retinoic acid (RA), as a requisite driver of spermatogonia differentiation and entrance into meiosis, regulates the PI3K/AKT/mTOR pathway to induce Kit translation during spermatogonial differentiation. In addition, Nanos2, an RNA-binding protein, interacts with mRNP to repress mTORC1 signaling by trapping mTOR, providing a post-transcriptional buffering system for SSCs homeostasis. Apart from germ cells, mTOR also plays a pivotal role in Sertoli cells. The regulation of Sertoli cell proliferation by FSH requires the PI3K/AKT/mTORC1 pathway. Independent of the canonical Rheb/mTORC1 pathway, Raptor dominates Sertoli cell cytoskeletal organization and polarity by affecting cingulin expression and Rac1 activity. Consistently, mTOR regulates gap junction alpha-1 (GJA1) distribution in Sertoli cells and is essential for progression through the pachytene spermatocyte stage. Finally, mTORC1 regulates spermatogenesis by inhibiting autophagy initiation and autophagosome nucleation. (**b**) Role of autophagy in spermiogenesis. In terms of the role of autophagy in spermiogenesis, autophagy-related genes participate in acrosome biogenesis, flagella assembly, head shaping and the removal of cytoplasm from elongating spermatids. 1. Acrosome biogenesis. TBC1D20 facilitates autophagy flux by its RAB1B GAP function, and regulates the formation of acrosome, which is a LRO. Sirt1 regulates spermiogenesis by stimulating autophagy. I. The depletion of *Sirt1* disrupts LC3 and ATG7 deacetylation, provoking the redistribution of LC3 from the nucleus to the cytoplasm. II. Golgi-derived vesicles fail to recruit LC3. And III. nucleus-associated acrosomal vesicles are unable to recruit GOPC and PICK1. ATG7 not only partially targets GOPC to control acrosome biogenesis, but also motivates LC3, which initiates autophagy. 2. Flagella assembly, head shaping, cytoplasm removal. ATG7 is required for spermatozoa flagella biogenesis and cytoplasm removal during spermiogenesis. IFT20, as a Golgi transport protein, contributes to the formation of autophagosome by delivering ATG16L, and lysosome biogenesis by regulating the post-Golgi transport of acid hydrolases. SPEF2 is elementary for microtubule-mediated transport in axonemal CP assembly and sperm head shaping. IFT20 interacts with SPEF2 to regulate the flagella. The autophagy-lysosome pathway regulates spermatid differentiation by degrading PDLIM1 to facilitate cytoskeleton organization. LRO, lysosome-related organelle; GOPC, Golgi-associated PDZ- and coiled-coil motif-containing protein; PDLIM1, PDZ and LIM domain 1; SPEF2, sperm flagellar 2.

mTORC1, as a central modulator in stem cell homeostasis (Yilmaz *et al.*, 2006; Chen *et al.*, 2008; Gan and DePinho, 2009), is critical for maintenance of the pool of SSCs (Serra *et al.*, 2019). Imbalances of SCC self-renewal and differentiation before meiosis can cause spermatogenesis disruption, even male infertility (Busada *et al.*, 2015b). Although germ cell conditional knockout (cKO) mice for mTORC1-specific component *Raptor* were viable and healthy, spermatogonial proliferation was reduced in the neonatal testis, and blocked in the juvenile and adult testis, suggesting that mTORC1 is autonomously required for SCC proliferation and differentiation (Serra *et al.*, 2017, 2019). Further, high phosphorylation of Raptor by raptor overexpression induced rapid growth of cultured SSCs, indicating that proliferation of SSCs requires phosphorylation of the mTORC1 component Raptor at Ser^863^ (Wang *et al.*, 2017b). A recent study shows that conditional ablation of *Raptor* causes infertility due to meiotic arrest and impaired inactivation of sex chromosomes in the male germline (Xiong *et al.*, 2017). Male germline cKO of *Rheb*, a critical component for mTORC1 activation, leads to defects of meiotic progression and spermiogenesis (Baker *et al.*, 2014). Collectively, mTORC1 is crucial for SCCs proliferation and differentiation meiotic progression, silencing of sex chromosomes and spermiogenesis (Fig.  3).

When it comes to the upstream and downstream signaling of mTORC1 in the spermatogenesis, Zhou *et al.* (2015) firstly confirm that Nanos2, an RNA-binding protein, interacts with messenger ribonucleoprotein (mRNP) to repress mTORC1 signaling by trapping mTOR, providing a post-transcriptional buffering system for SSCs homeostasis. Meanwhile, GILZ is indispensable for the modulation of mTORC1 in SSCs (La *et al.*, 2018). Lin28a promotes SSCs proliferation through regulating mTOR and PI3K/AKT in dairy goats (Ma *et al.*, 2016). Retinoic acid (RA), as a requisite driver of spermatogonial differentiation and meiosis, regulates the PI3K/AKT/mTOR pathway to induce Kit translation during spermatogonial differentiation in mice (Busada *et al.*, 2015a).

Apart from the role in germ cells, mTOR also plays a pivotal role in Sertoli cells. The regulation of Sertoli cell proliferation by FSH requires the PI3K/AKT/mTORC1 pathway (Riera *et al.*, 2012). Loss of the mTORC1 component *Raptor* in Sertoli cells leads to severe tubular degeneration in the neonatal testis, azoospermia and cytoskeletal organization disruption in adult mice (Xiong *et al.*, 2018). Independent of the canonical Rheb/mTORC1 pathway, Raptor dominates Sertoli cell cytoskeletal organization and polarity by affecting cingulin expression and Rac1 activity (Xiong *et al.*, 2018). Sertoli cell cKO mice for *Mtor* exhibit similar phenotypes, but a further study demonstrates that mTOR regulates gap junction alpha-1 (GJA1) distribution in Sertoli cells and is essential for progression through the pachytene spermatocyte stage (Boyer *et al.*, 2016). Given that the mTORC2 component Rictor also controls spermatogonial differentiation and intercellular adhesion (Bai *et al.*, 2018), the functions of mTORC1 and mTORC2 overlap partially. mTORC1 and mTORC2 may synergistically orchestrate blood–testis barrier (BTB) dynamics by intercellular adhesion and cytoskeleton. To check this assumption, much work is needed to investigate whether mTOR complexes exert their effects on the F-actin via drebrin E, paladin, formins, filamins, Eps8, the Arp2/3 complex or others (Mok *et al.*, 2013).

Our previous research demonstrated that the PI3K inhibitor 3-MA rescues apoptosis by partially aggravating the reduction of the autophagy flux in cadmium-treated mouse spermatogonia, while in cadmium-treated mouse spermatocyte cells, 3-MA rescued apoptosis by inhibiting autophagy (Wang *et al.*, 2020). The results imply that autophagy exerts different effects on spermatogonial cells and spermatocyte cells in response to external stimuli. Actually, in PSC, autophagy acts as a protective mechanism for mitochondrial abnormalities induced by *Trl* gene mutations (Dorogova *et al.*, 2014). In spermatogonia, except from pro-survival function, autophagy implements a pro-death function.

Mancilla *et al.* (2015) demonstrated that deleting glutathione (GSH)-induced autophagy promotes cell survival by antagonizing apoptosis via the AMPK-independent pathway in the mouse spermatogonia cell line, GC-1 cells. Similarly, autophagy plays a cytoprotective role in nutrient-deprived GC-1 cells by the ANKRD49 and NF-κB pathways (Wang *et al.*, 2015). Instead, Tri-ortho-cresyl phosphate (TOCP)-mediated autophagy contributes to cell death. TOCP, as widespread plasticizers and flame retardants, have been known to induce testicular toxicity since 1987 (Somkuti *et al.*, 1987). Recently, TOCP was further reported to decrease the viability of spermatogonia and motivate autophagy, which was confirmed by increases in LC3-II/LC3-I, ATG5 and BECN1 in mice and rats (Chen *et al.*, 2012; Liu *et al.*, 2015). Intriguingly, the cell cycle and apoptosis present no noticeable changes (Liu *et al.*, 2015). According to the three updated criteria for identifying autophagic cell death as proposed by Shen and Codogno (2011), the first criterion is that apoptosis is not involved in the issue. Therefore, it is reasonable to suspect that autophagy contributes to autophagic cell death, which is constitutes a pro-death mechanism in TOCP-induced spermatogonia injury. Nonetheless, in terms of this study, more experimental evidence, such as the activation of an autophagy flux and rescue by an autophagy inhibitor, is required for verifying these speculations.

### Role of autophagy in spermatidogenesis

During spermatidogenesis, each diploid PSC develops into four haploid round spermatids through meiosis, which occupies an absolute central position in the process. One PSC undergoes one round of DNA replication and cell division (Meiosis I) to produce two haploid secondary spermatocytes, which subsequently proceed through the second cell division (Meiosis II) to produce four equal haploid round spermatids (Wang *et al.*, 2017a). Meanwhile, a series of distinctive cellular events occur, including programmed DNA double-strand break formation, homologous recombination, crossover formation and resolution (Sung and Klein, 2006).

The study on the role of autophagy in spermatidogenesis originates in 1986. Chemes (1986) proved that Sertoli cells phagocytized and digested meiotic spermatocyte residual bodies by autophagy in a testosterone-independent manner. Recent research showed that autophagy and apoptosis are synchronously provoked in the heat-treated mouse spermatocyte cell line, GC-2 cells. Dramatically, Atg7-mediated downregulation of autophagy reduced apoptosis in heat-induced GC-2 cells, indicating that autophagy and apoptosis act as partners to promote cell death (Zhang *et al.*, 2012).

Apart from being pro-death, autophagy seems to undertake a dual mission in regulating chromatoid bodies (CBs) during spermatidogenesis. CBs are a typical cytoplasmic features of haploid round sperm cells, consisting of RNA and RNA-binding proteins, and are unique ribonucleoprotein (RNP) granules (Meikar *et al.*, 2011). Despite appearing in late pachytene spermatocytes, the CB-like granules are immediately condensed into a single granule after meiosis and maintain their character throughout the differentiation of round spermatids (Kotaja and Sassone-Corsi, 2007). Due to the accumulation of plentiful PIWI-interacting RNA (piRNA) in the CBs of round spermatids, the CB is deemed to be responsible for piRNA-targeted RNA regulation (Gomes Fernandes *et al.*, 2018). Remarkably, piRNA-directed cleavage of meiotic transcripts regulates spermatogenesis (Goh *et al.*, 2015). Therefore, it is rational to suspect that CBs govern meiotic transcripts during spermatidogenesis.

Interestingly, Da Ros *et al.* (2017) discovered that the LC3B-interacting protein FYCO1, a novel CB component, initiates the intracellular transport of autophagic vesicles, which regulates the integrity of RNP granules in haploid male germ cells. Furthermore, both the agonists and antagonists of autophagy aggravate the cellular defects (fragmented CB) in the *Fyco1* cKO germ cells, manifesting that autophagy devotes itself to two distinct events: the clearance of CB materials and the maintenance of CB homeostasis, synchronously (Da Ros *et al.*, 2017). Consistent with the perception, autophagy can protect genomic stability by degrading retrotransposon RNA (Guo *et al.*, 2014), and PIWI proteins/piRNA in the CB are the targets of degradation after autophagy activation (Siomi *et al.*, 2011; Da Ros *et al.*, 2017). Taken together, autophagy plays three roles during spermatidogenesis: (i) pro-death in spermatocyte, (ii) clearance of CB materials in round spermatids and (iii) maintenance of genomic stability in CBs by degrading PIWI proteins/piRNA in round spermatids.

The regulation of spermatidogenesis by autophagy was confirmed in *Ppp1cc* and *Raptor* KO mice, respectively. *Ppp1cc2*, as a substrate of the CMA regulator Hsc70 (Bonam *et al.*, 2019; Itoh *et al.*, 2019), was required for chromatin condensation and acrosome development (Forgione *et al.*, 2010). *Ppp1cc* KO mice exhibited meiosis arrest during spermatidogenesis and spermiogenesis, presenting polyploid spermatids (Varmuza *et al.*, 1999). Similarly, conditional ablation of mTORC1 component Raptor in the male germline caused meiotic arrest (Xiong *et al.*, 2017). Therefore, the cross-talk between meiosis and autophagy-related proteins needs further exploration during spermatidogenesis.

### Role of autophagy in spermiogenesis

Spermiogenesis refers to the final stage of spermatogenesis when round spermatids become elongated and then develop into spermatozoa through drastic morphological changes. Spermiogenesis involves the reshaping of the nucleus, rearrangement of mitochondria and development of flagellum and acrosome (Tanaka and Baba, 2005). These orchestrated physiological processes require a cellular homeostasis between degradation and recycling of cytoplasmic components. Recently, growing evidence indicates that autophagy, as a unique catabolic pathway (Ktistakis and Tooze, 2016), is involved in spermiogenesis. Autophagy-related proteins (LC3, ATG5, ATG16, BECN1, p62, mTOR, AMPKα 1/2 and PINK1) and their upstream regulators are functionally active in human spermatozoa, suggesting that autophagy may regulate sperm motility (Aparicio *et al.*, 2016). Specifically, LC3 and ATG7 are increased dramatically from the round to elongated spermatids (Yang *et al.*, 2017). Besides, autophagosomes in spermatozoa originate from the bilayer separation membrane of the chrysanthemum flower center, which is converted from endoplasmic reticulum (Yang *et al.*, 2017), implying the regulation of spermiogenesis by autophagy.

The acrosome is also a unique lysosome-related membranous organelle (MO) in the anterior part of the sperm nucleus (Berruti and Paiardi, 2011). The acrosome carries hydrolytic enzymes to facilitate sperm penetrating the zona pellucida (Jin *et al.*, 2011; Ozturk *et al.*, 2017). For the first time, Hartree proposed the correlation between the acrosome and the lysosome in 1975 (Hartree, 1975). Controversially, Martínez-Menárguez et al. argued in 1996 that acrosomes were independent of endosomes or lysosomes due to the absence of late endosomal marker cation-dependent and non-dependent mannose 6-phosphate receptor in acrosomal vesicles and preantral vesicles (Martinez-Menarguez *et al.*, 1996). Ten years later, both Berruti *et al.* (2010) and Hu *et al.* (2007) demonstrated that the acrosome was indeed a lysosome-related organelle (LRO). Furthermore, acrosome biogenesis, as a hinge of spermiogenesis (Kang-Decker *et al.*, 2001), was linked closely with autophagy.

Generally, acrosome biogenesis is divided into four major phases: Golgi, cap, acrosome and maturation phases (Khawar *et al.*, 2019). The first phase corresponds to the perspective that acrosome is derived from Golgi (Berruti and Paiardi, 2011). Research verifies that Golgi can be regulated by TBC1D20 (Haas *et al.*, 2007), and the disruption of TBC1D20 results in testicular abnormalities in mice (Park *et al*., 2014), as well as in humans (Liegel *et al.*, 2013). Thus, TBC1D20 may mediate the testicular function via regulating Golgi. TBC1D20, as a member of GAPs, interacts with GTP and subsequently, the ‘active’ GTP-bound RAB-GTPase is returned to the ‘inactive’ GDP-bound state (Frasa *et al.*, 2012). Strikingly, Sidjanin *et al.* (2016) showed that TBC1D20 facilitates autophagy flux by its RAB1B GAP function, and regulates the formation of acrosome in mice, suggesting that TBC1D20 may regulate acrosome biogenesis via autophagy.

In parallel, *Sirt1* regulates acrosome biogenesis by modulating autophagy flux during spermiogenesis in mice. Liu *et al.* found that the depletion of *Sirt1* undermined spermiogenesis by stimulating autophagy in mice spermatids, including three successional cellular events: (i) the depletion of Sirt1 disrupted LC3 and ATG7 deacetylation, leading to the redistribution of LC3 from the nucleus to the cytoplasm; (ii) Golgi-derived vesicles failed to recruit LC3; and (iii) nucleus-associated acrosomal vesicles were unable to recruit Golgi-associated PDZ- and coiled-coil motif-containing protein (GOPC) and PICK1 (Liu *et al.*, 2017a). In addition, for this comprehensive research, further studies proved that ATG7 not only partially targeted GOPC to control acrosome biogenesis (Wang *et al.*, 2014), but is also essential for activation of LC3, which initiated autophagy (Tanida *et al.*, 2012). Even, ATG7 was required for spermatozoa flagella biogenesis and cytoplasm removal during mice spermiogenesis (Shang *et al.*, 2016). In terms of the mechanism, research elucidated that the autophagy-lysosome pathway regulates spermatid differentiation by degrading negative cytoskeleton regulator PDZ and LIM domain 1 (PDLIM1) to facilitate cytoskeleton organization (Shang *et al.*, 2016). Coincident with this assertion, in moss, ATG5- and ATG7-mediated autophagy promote the flagellated motile sperm differentiation and cytoplasmic elimination (Sanchez-Vera *et al.*, 2017). Therefore, whether in mice or moss, autophagy is indispensable for spermatid differentiation, especially in acrosome biogenesis and flagella biogenesis.

When it comes to flagella biogenesis, it is important to mention intraflagellar transport 20 (IFT20). During mouse spermiogenesis, IFT20, as a Golgi transport protein, interacts with the sperm flagellar 2 (SPEF2) to regulate the development of the sperm flagella (Lehti *et al.*, 2017). SPEF2 is critical for microtubule-mediated transport in axonemal CP assembly and sperm head reshaping in mouse (Lehti *et al.*, 2017). Consistently, in humans, homozygous mutations in *SPEF2* lead to multiple morphological abnormalities of the sperm flagella and male infertility (Liu *et al.*, 2020). These solid data support that IFT20 and SPEF2 are required for sperm tail formation and head reshaping during spermiogenesis. Although LC3 and ubiquitin are well-balanced in the testis of the *Ift20* mutant mice (Zhang *et al.*, 2016), it is not enough to deny the correlation between IFT20 and autophagy because of the scarcity of autophagy flow evaluation in this limited study. Remarkably, IFT20 contributes to autophagosome formation by delivering ATG16L (Pampliega *et al.*, 2013) and to lysosome biogenesis by regulating the post-Golgi transport of acid hydrolases (Finetti *et al.*, 2020).

Hence, autophagy participates in acrosome biogenesis, flagella assembly, shaping of the head and the removal of elongating spermatid cytoplasm during spermiogenesis (Fig.  3).

### Role of autophagy in spermiation

Spermiation is the release of elongated spermatids from Sertoli cells into the seminiferous tubule lumen prior to the epididymis (Cheng and Mruk, 2010). This process is guided by a testis-specific, actin-based anchoring junction, apical ES (aES) in the Sertoli cell-spermatid interface (Mruk *et al.*, 2008). Apart for spermatids movement and release, aES also contributes to shaping the spermatid head (Toyama *et al.*, 2003; Mruk and Cheng, 2004). To achieve this goal, aES needs to undergo tightly and timely managed restructuring (Berruti and Paiardi, 2014).

**Figure 4.**
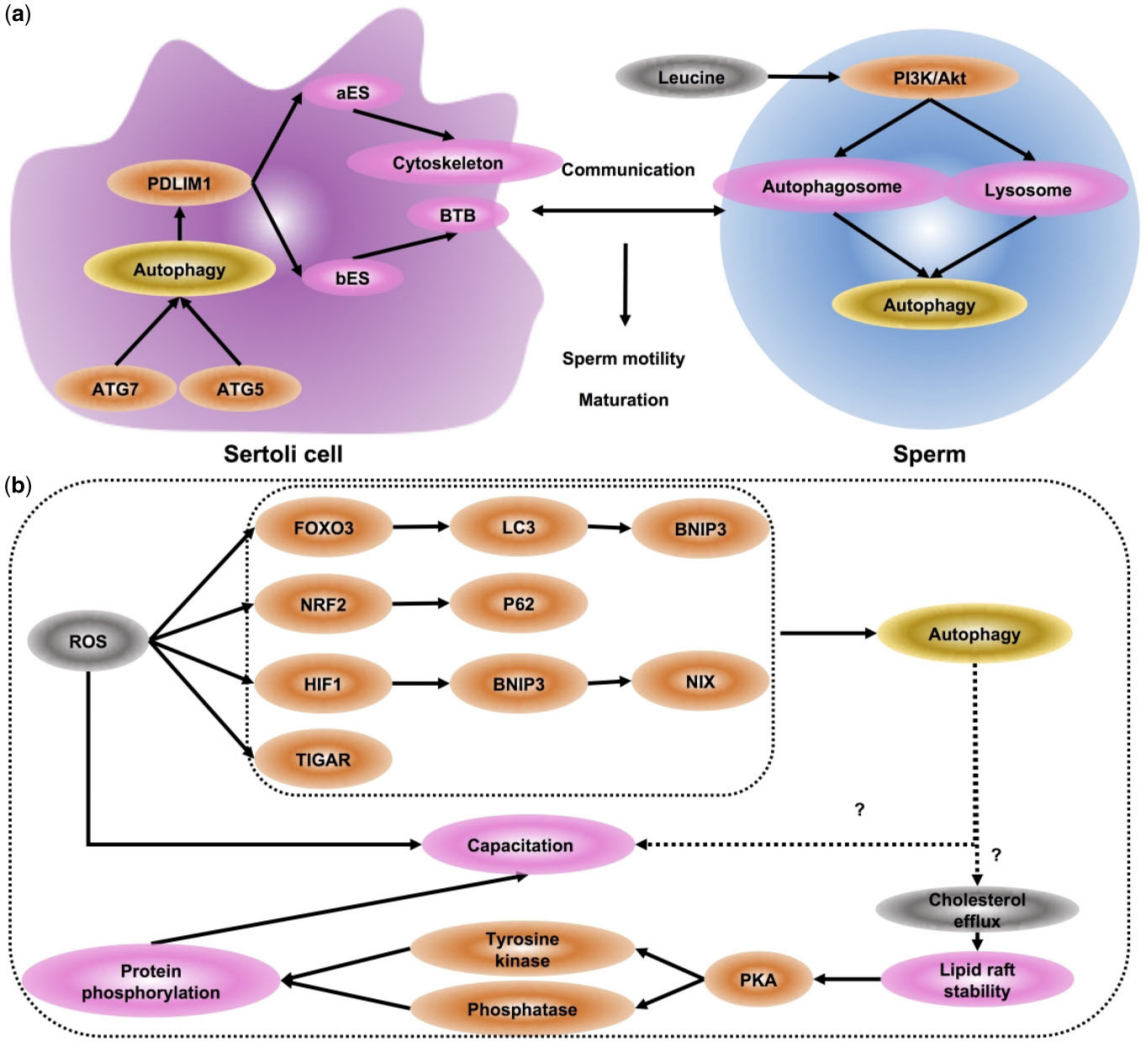
**Role of autophagy in sperm maturation.** (**a**) Sertoli cells. Cell-specific knockout of *Atg5* or *Atg7* results in spermatozoa with malformed heads and low motility. Through PDLIM1, autophagy not only affects bES to regulate BTB, but also mediates aES to handle cytoskeleton, governing spermatids movement and release during spermiation. In sperm: autophagy can affect sperm motility by inhibiting the fusion of autophagosome and lysosomes through the PI3K/Akt-dependent pathway in leucine-treated zebrafish. (**b**) Capacitation. ROS regulates autophagy by transcriptional and post-transcriptional regulation, including ROS-FOXO3-LC3/BNIP3-autophagy, ROS-NRF2-P62-autophagy, ROS-HIF1-BNIP3/NIX-autophagy and ROS-TIGAR-autophagy. Cholesterol efflux alters lipid raft stability during capacitation. PKA can stimulate protein phosphorylation and capacitation or acrosomal exocytosis by activating tyrosine kinase and/or inhibiting protein phosphatase. Nonetheless, so far, there is no direct evidence that autophagy participates in the process of capacitation. It may be a good potential target for exploring the molecular mechanisms in capacitation disruption-induced male infertility. PDLIM1, PDZ and LIM domain 1; aES, apical ectoplasmic specialization; bES, basal ectoplasmic specialization; BTB, blood–testis barrier.

Currently, autophagy has been demonstrated to be required for ES assembly. ES encompasses two parts: (i) aES, at the Sertoli cell-spermatids interface and (ii) basal ES (bES), at the Sertoli–Sertoli cell interface (Cheng and Mruk, 2010, 2012). Sertoli cell-specific KO of Atg5 or Atg7 results in spermatozoa with malformed heads and low motility, affecting the fertility of male mice (Liu *et al.*, 2016). Meanwhile, defective autophagy in Sertoli cells perturbs the degradation of PDLIM1, which is vital for cytoskeleton assembly (Liu *et al.*, 2016). Consequently, through PDLIM1, autophagy not only affects bES to regulate BTB, but also mediates aES to govern spermatids movement and release during spermiation (Fig.  4a).

## Post-testicular processes

### Role of autophagy in sperm maturation

For fertilization, elongated spermatids have to undergo maturation. Sperm maturation contains three highly orchestrated processes: (i) obtaining motility and fertility in the epididymis, (ii) capacitation in the female reproductive tract and (iii) activation upon the approach of ovary and sperm.

Firstly, sperm flows into the rete testis in Sertoli cell-secreted fluid. With the action of their flagella and smooth muscle contraction, sperm then enter the caput epididymis. In the epididymis, sperm maturation involves both changing flagellum beat and acquiring the characteristics necessary for effective contact with the oocyte (Sullivan and Mieusset, 2016). The sperm cannot attain fertilization ability until they reach the proximal end of the epididymis. The motility and fertility capacities of sperm are completed in the cauda epididymis.

Secondly, capacitation, as the penultimate step of mammalian sperm maturation, occurs in the female reproductive tract. When semen is ejaculated into a female vagina, the sperm leaves the semen and enters the uterine cavity and fallopian tube via the cervical canal. Then female genital tract secrets alpha and beta-amylase, which degrades the acrosome surface glycoprotein. Meanwhile, the sperm launches a series of orchestrated biochemical reactions, including phosphorylation, alkalinization and hyperpolarization, dependent on CFTR and PKA, preparing for penetrating out layer of the oocyte (Puga Molina *et al.*, 2017). Additionally, the increase in Ca2+ influx allows the sperm tail greater motility (Ren *et al.*, 2001). Finally, capacitation renders sperms competent to fertilize an oocyte.

Notably, a dramatic change of ROS occurs in sperm during capacitation (Fig.  4b). Despite the detrimental effect of high concentrations of ROS, low concentrations of ROS are beneficial for sperm fertilization. It is pivotal to balance the homeostasis between ROS and autophagy. ROS regulates autophagy by transcriptional and post-transcriptional regulation, including ROS-FOXO3-LC3/BNIP3-autophagy, ROS-NRF2-P62-autophagy, ROS-HIF1-BNIP3/NIX-autophagy and ROS-TIGAR-autophagy (Li *et al.*, 2015). In turn, autophagy can regulate ROS levels through CMA, the mitotic pathway and the P62 delivery pathway (Li *et al.*, 2015). Also, autophagy can affect sperm motility by inhibiting the fusion of autophagosomes and lysosomes through the PI3K/AKT-dependent pathway in leucine-treated zebrafish (Zhang *et al.*, 2017).

Given that cholesterol efflux alters lipid raft stability and distribution during capacitation of boar spermatozoa (Shadan *et al.*, 2004), cholesterol efflux may have close relationship with capacitation. Recent studies have shown a strong link between the PKA pathway and sperm capacitation. PKA can stimulate protein phosphorylation and capacitation or acrosomal exocytosis by activating tyrosine kinase and/or inhibiting protein phosphatase (Puga Molina *et al.*, 2017).

Nonetheless, so far, there is no direct evidence that autophagy participates in the process of capacitation (Fig.  4b). It may be a good potential target for exploring the molecular mechanism in capacitation dysfunction-induced male infertility.

### Role of autophagy in fertilization

Fertilization is a fundamental process in reproduction (Ohto *et al.*, 2016). In this process, the oocyte and sperm achieve mutual recognition, and fuse into a zygote, which then develops into a new individual, allowing for the continuity of a species (Okabe, 2013).

Classically, the acrosome reaction is one of the most critical steps in fertilization. This may be due to the fact that acrosome reaction allows the sperm to release acrosomal enzymes, dissolve the radiation corona and penetrate the zona pellucida (Tosti and Menezo, 2016). Recent research shows that NAADP and the two-pore channel (TPC) protein 1 participate in the acrosome reaction by Ca2+ release in mammalian spermatozoa (Arndt *et al.*, 2014). The regulation of TPCs by autophagy is involved in multiple cells, such as neural cells (Pereira *et al.*, 2017), cardiomyocytes (Garcia-Rua *et al.*, 2016) and cancer cells (Sun and Yue, 2018). TPC2 mediates mTORC1 (Chang *et al.*, 2020), which is the main gateway to autophagy (Rabanal-Ruiz *et al.*, 2017). In turn, mTORC1 controls lysosomal Ca2+ release by TPC2 (Ogunbayo *et al.*, 2018). Despite the interaction of TPC2 and mTORC1, there is no direct evidence that autophagy regulates the acrosome reaction through TPCs. This is waiting to be clarified.

During fertilization, most animals only inherit the mitochondrial genome from the maternal parent (Ankel-Simons and Cummins, 1996). The paternal mitochondria are eliminated in the embryo, although the sperm provides DNA, centrioles, some cytoplasm and organelles for the offspring (Kaneda *et al.*, 1995). The underlying mechanism of PME during fertilization remains unclear.

Fertilization-triggered autophagy can degrade paternal mitochondria (Sato and Sato, 2011; Zhou *et al.*, 2011), and post-fertilization autophagy of sperm organelles prevents paternal mitochondrial DNA (mtDNA) transmission in *Caenorhabditis* *elegans* (Al Rawi *et al.*, 2011). Zhou *et al.* (2016) found that CPS-6 migrated from the parent mitochondrial intermembrane to the matrix after fertilization for the degradation of mtDNA. Loss of cps-6 disrupts autophagy, PME and embryogenesis, revealing that CPS-6 regulates PME by interacting with maternal autophagy and proteasome machinery upon fertilization in *C. elegans* (Zhou *et al.*, 2016). Tsukamoto *et al.* discovered that autophagy-deficient sperm (sperm-specific KO ATG5) and oocytes without ATG5 in mice could undergo regular fertilization and fuse into a zygote, but failed to develop into four-cell and eight-cell stages. In contrast wild-type sperm and autophagy-deficient oocytes were available to develop normally into embryos, suggesting that fertilization-triggered autophagy was essential for the development of mammalian early embryos (Tsukamoto *et al.*, 2008).

In mice embryos, this autophagic degradation process is dependent on the E3 ubiquitin ligases PARKIN and MUL1, mitochondrial outer membrane protein FIS1, autophagy adaptor p62 and PINK1 kinase (Rojansky *et al.*, 2016). Owing to mitochondrial depolarization stabilizing PINK1, both PINK1 and PARKIN are recruited to damaged mitochondria, and latent PARKIN is activated for mitophagy in Parkinson’s disease (Kawajiri *et al.*, 2010; Matsuda *et al.*, 2010). PINK1/PARKIN-mediate mitophagy through voltage-dependent anion channel 1 and p62/SQSTM1 (Geisler *et al.*, 2010). Although in *Drosophila*, autophagy and endocytosis regulate PME by a PARKIN-independent pathway (Politi *et al.*, 2014), PINK1/PARKIN-mediated mitophagy may be essential for PME in mammals.

Another study demonstrated that fertilization induces ubiquitination and recruitment of LGG-1/LGG-2 (the two LC3 homologs in worms) around the flagellum mid-piece; after fertilization, paternal mitochondria and MOs are engulfed in autophagosomes and degraded during the first zygote divisions (Al Rawi *et al.*, 2012). Furthermore, the depletion of lgg-1 led to the death of 95% animals at or before the L1 larval stage, implying that disruption of PME may undermine embryogenesis, although there were other zygotic defects due to the loss of LGG-1 (Levine and Elazar, 2011; Sato and Sato, 2011). Djeddi *et al.* (2015) verified that sperm-inherited organelle clearance in *C. elegans* relied on LC3-dependent autophagosomes. The above results suggest that both proteasomal and autophagic degradation participate in PME and MOs clearance upon fertilization in *C. elegans*.

In parallel, Hajjar *et al.* confirmed that sperm-derived mitochondria and MOs, which cluster together, are simultaneously decreased during fertilization in *C. elegans*. K48-linked ubiquitin chains (K48 chains), which degrade proteasomes, arise on MOs and vanish very quickly; meanwhile, K63-linked ubiquitin chains (K63 chains), which recruit autophagosomal markers (LGG-1/2) to MOs, emerge on MOs early and remain throughout the first several cell divisions (Hajjar *et al.*, 2014). These findings suggest that K63 chain-mediated autophagy modulates PME and MOs clearance, and K48 chains only participate in MOs clearance. However, the interaction mechanism between K63 chains and K48 chains in PME or MOs clearance remains an enigma.

Song *et al.* (2016) showed that sperm mitophagy in pig and rhesus monkey relied on p62-dependent autophagy, valosin-containing protein-mediated dislocation and recruitment of ubiquitin to the 26S proteasome. They concluded that autophagy and the UPS contributed to sperm mitophagy after mammalian fertilization (Song *et al.*, 2016).

Interestingly, Al Rawi *et al.* (2011) and Sato and Sato (2011) claim that ubiquitin was only related to MOs but not paternal mitochondria, despite the fact that autophagosomes indeed degrade both MOs and paternal mitochondria. In view of this, Levine and Elazar put forward two potential roles of paternal MOs by suggested that (i) MOs provided membranes for autophagosome formation and (ii) MOs launched autophagy in the process of PME (Levine and Elazar, 2011).

However, this theory was challenged by Luo *et al.* (2013). They proposed that autophagy was not involved in PME after fertilization by using two transgenic mice, separately labeling mitochondria and LC3, although some autophagy-related proteins, such as p62/SQSTM1 and LC3 indeed localized near the sperm mitochondria before the two-cell stage. Additionally, most motile sperm eliminated their mtDNA before fertilization (Luo and Sun, 2013). Thus, in mice, maternal inheritance of mtDNA may be a passive process as a result of pre-fertilization sperm mtDNA elimination and uneven mitochondrial distribution in embryos (Luo and Sun, 2013). Whether this is the case awaits further research.

**Figure 5.**
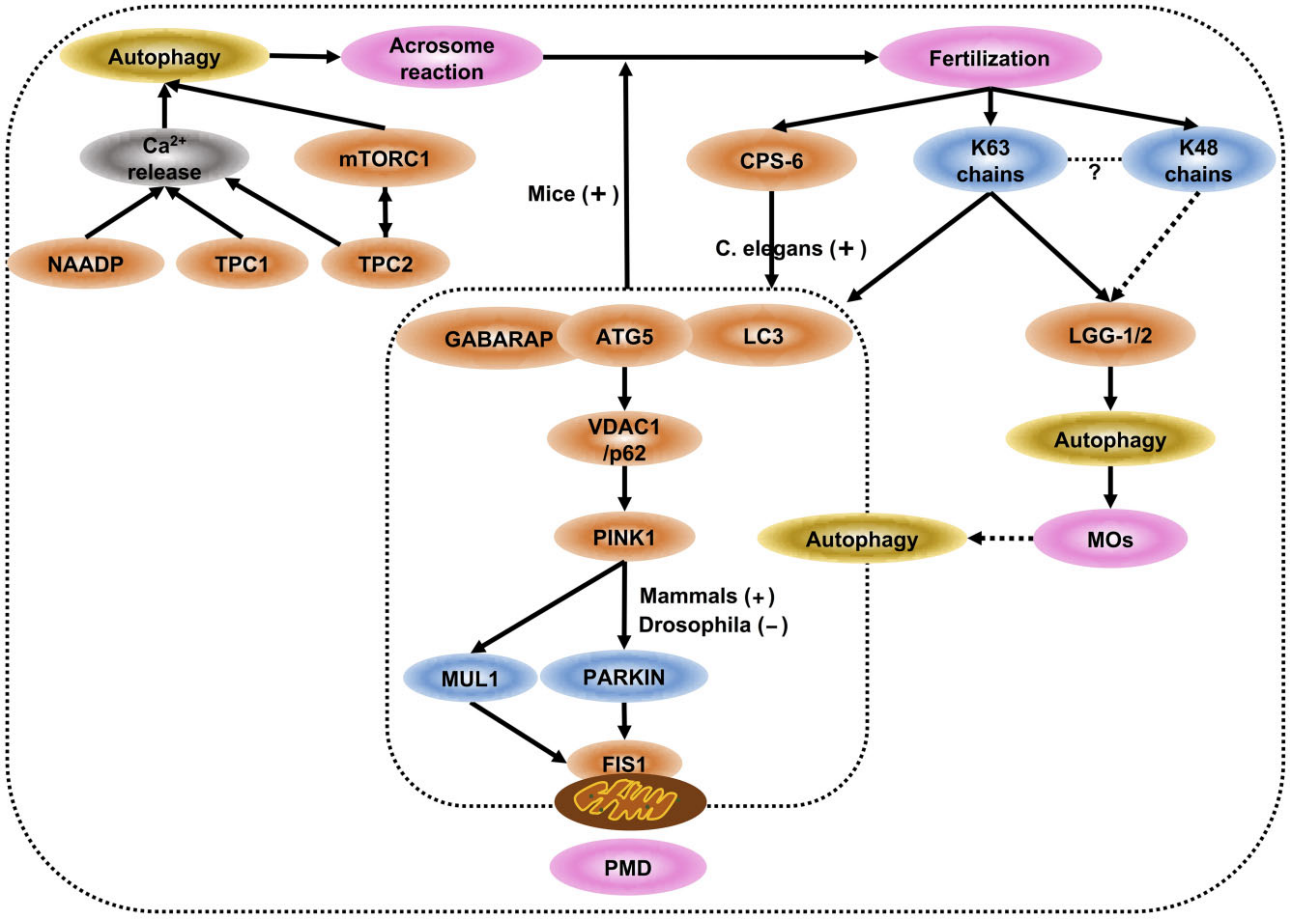
**Role of autophagy in fertilization.** Autophagy regulates the acrosome reaction, PME and MOs clearance during fertilization. (**a**) Acrosome reaction. NAADP and the two-pore channel (TPC) protein 1 participate in the acrosome reaction by Ca2+ release in mammalian spermatozoa. TPC2 mediates mTORC1, which is the main gateway to autophagy; in turn, mTORC1 controls lysosomal Ca2+ release by TPC2. Autophagy may operate the acrosome reaction through TPCs. (**b**) PME and MOs clearance. Both autophagy and the ubiquitin-proteasome system contribute to PME and MOs after fertilization. In *C. elegans*, CPS-6 regulates PME, and LGG-1/LGG-2 (LC3 homologs) operate PME and MOs clearance by interacting with maternal autophagy and proteasome machinery upon fertilization; K63 chains-mediated autophagy may modulate PME and MOs clearance, and K48 chains may participate in MOs clearance. However, the crosstalk of K63 chains and K48 chains remains an enigma. In mice, fertilization-triggered autophagy regulates the development of mammalian early embryos by ATG5; this autophagic degradation process is dependent on the E3 ubiquitin ligases PARKIN and MUL1, mitochondrial outer membrane protein FIS1, autophagy adaptor p62 and PINK1 kinase. Ubiquitin is only related to MOs but not PME, despite the fact that autophagosomes degrade both of MOs and paternal mitochondria. Two potential roles of paternal MOs are that MOs provide membranes for autophagosome formation or MOs launch autophagy through the process of PME. Autophagy is not involved in PME after fertilization: in mice, maternal inheritance of mtDNA may be a passive process as a result of pre-fertilization sperm mtDNA elimination and uneven mitochondrial distribution in embryos. The actual situation awaits further research. PME, paternal mitochondrial elimination; MOs, membranous organelles; TPC, the two-pore channel; mtDNA, mitochondria DNA.

To sum up, autophagy may regulate the acrosome reaction, PME and MOs clearance during fertilization (Fig.  5).

## Clinical impact of autophagy on the fate of sperm

Dysfunctional autophagy contributes to many diseases, which determine the clinical impacts of autophagy. Currently, autophagy activators (such as rapamycin, perifosine, metformin) and autophagy inhibitors (such as bafilomycin A1, CQ, lucanthone, wortmannin) have been investigated for clinical and translational medicine (Table  IV) and present good prospects in clinical application.

**Table IV:** The clinical prospects of autophagy activators and inhibitors.

Drugs	Diseases	Functions	Possible mechanism	In human/animal/cell model	Reference
**Autophagy activators**					
**Rapamycin**	Breast cancer	Inhibits proliferation of the endoplasmic reticulum-positive MCF-7 cell line	Rapidly stimulate mTOR non-specifically after medium replacement	MCF-7 breast cancer cell line	Chang *et al.* (2007)
	Transplanted tumors	Inhibits tumor growth at any stage of development	–	–	Eng *et al.* (1984)
	Pancreatic cancer	Regulates cell growth and cyclin D1 expression	Constitutively actives FRAP-p70s6K pathway and inhibits cyclin D1 expression	MiaPaCa-2 and Panc-1 human pancreatic cancer cells and a pancreatic cancer tissue sample	Grewe *et al.* (1999)
	Vascular disease	Reduces vascular inflammation	Suppresses macrophage proliferation	Mice	Boada *et al.* (2020)
	Type 2 diabetes	Improves insulin resistance and hepatic steatosis	Enhances autophagy by the inhibition of mTOR pathway	T2DM rats	Zhou and Ye (2018)
	Myotrophic lateral sclerosis	Reduces neuronal loss and TDP43 inclusions; expands regulatory T lymphocytes with slow progression in ALS patients	Activates autophagy	Four human NB cell lines (AS, NGP, BE2, and KCNR); mice carrying xenograft NB tumors	Mandrioli *et al.* (2018)
	Facial angiofibromas	Appears effective and safe for treatment of TSC-related facial angiofibromas	–	TB3 cells	Koenig *et al.* (2018)
		Improves ovarian function and reproductive longevity	–	–	Hine (2017)
**Perifosine**	Neuroblastoma	increases apoptosis and inhibits neuroblastoma tumor cell growth *in vitro* and *in vivo*	Decreases AKT phosphorylation	Four human NB cell lines (AS, NGP, BE2, and KCNR); mice carrying xenograft NB tumors	Li *et al.* (2010)
	Neuroblastoma	Attenuates brain-derived neurotrophic factor/TrkB-induced chemoresistance	Inhibits AKT	TB3 cells	Li *et al.* (2011)
**Metformin**	Glioblastoma	Inhibits growth of human glioblastoma cells and enhances therapeutic response	Activates AMPK, Redd1 and inhibits mTOR pathway	Four human glioblastoma cell lines, U87 (ATCC HTB-14), LN18 (ATCC CRL-2610), U251 and SF767	Sesen *et al.* (2015)
**Metformin and Rapamycin**	Prostate tumors	Inhibits progression of prostatic intraepithelial neoplasia lesions to adenocarcinomas in the ventral prostate	Down-regulates mTORC1 signaling	HiMyc mice	Saha *et al.* (2015)
**Metformin**	Reproductive health, gynecological cancer	Inhibits progression of prostatic intraepithelial neoplasia lesions to adenocarcinomas in the ventral prostate	Upstream activation of AMPK, resulting in inhibition of the mTOR pathway.	HiMyc mice	Saha *et al.* (2015)

**Autophagy inhibitors**					
**Bafilomycin A1**	Pediatric B-cell acute lymphoblastic leukemia	Inhibits and kills pediatric B-cell acute lymphoblastic leukemia cells	Targets both autophagy and apoptosis by disassociating the Beclin 1–Vps34 complex	–	Yuan *et al.* (2015)
	Microcephaly	Inhibits ZIKV entry and prevents the spread of the infection by interfering with viral maturation	Inhibits V-ATPase	–	Sabino *et al.* (2019)
	Tongue squamous cell carcinoma	Increases the sensitivity of tongue squamous cell carcinoma cells to cisplatin	Inhibition of the lysosomal uptake of platinum ions but not autophagy	–	Chu *et al.* (2018)
**Chloroquine**	Breast cancer	Enhances the efficacy of tumor cell killing by combination with chemotherapeutic drugs and radiation	–	–	Maycotte *et al.* (2012)
	Colon cancer	Enhances the chemotherapeutic activity of 5-fluorouracil in a colon cancer cell line via cell cycle alteration	Anti-cancer effect of 5-FU via cell cycle inhibition	Human colon cancer DLD-1 cells	Choi *et al.* (2012)
**3-MA or chloroquine**	Glioblastomas	Improves the efficacy of curcumin/temozolomide combination therapy	Increases apoptosis	C6, U251MG and U87MG cell lines; primary astrocytes	Zanotto-Filho *et al.* (2015)
	Malignant gliomas	Enhances temozolomide cytotoxicity	Blocks autophagy and triggers endoplasmic reticulum stress, increasing the chemosensitivity of glioma cells to temozolomide	Subcutaneously implanted U87MG tumors from mice	Golden *et al.* (2014)
	Glioma	Potentiates temozolomide cytotoxicity	Inhibits mitochondrial autophagy	Tumor cells derived from a glioblastoma patient and human U87-MG glioblastoma cells	Hori *et al.* (2015)
**Hydroxy chloroquine**	Advanced solid tumors and melanoma	Augments cell death in preclinical models	Blocks autophagy	–	Rangwala *et al.* (2014a,b)
**Lucanthone**	Breast cancer	Induces apoptosis via cathepsin D accumulation and enhances vorinostat-mediated cell death in breast cancer models.	Induces lysosomal membrane permeabilization	p53(+/+) and p53(−/−) HCT116 cells	Carew *et al.* (2011)
**Wortmannin**	Ovarian cancer	Enhances cisplatin-induced apoptosis	Activates PI3K/Akt signaling pathway	A2780 ovarian adenocarcinoma cell line and A2780cis	Zhao *et al.* (2014)

3-MA, 3-methylade nine; AKT, protein kinase B; ALS, amyotrophic lateral sclerosis; AMPK, AMP-activated protein kinase; MCF-7 cells, human breast cancer cell lines; mTOR, mammalian target of rapamycin; NB tumor, neuroblastoma; T2DM rat, type 2 diabetes mellitus rat; TrkB, neurotrophic receptor tyrosine kinase 2; TSC, tuberous sclerosis complex; ZIKV, Zika virus.

It has been clearly shown that autophagy could be associated with infertility, in particular for patients with cryptorchidism (Yefimova *et al.*, 2019). Cryptorchidism, i.e. undescended testis, is recognized as one of the strongest risk factors for infertility in adulthood (Gurney *et al.*, 2017). Autophagy is increased in cryptorchid testis resulting in abnormal spermatozoa, thus autophagy maybe a potential target to improve sperm quality in cryptorchid men (Yefimova *et al.*, 2019).

During pre-testicular processes, the dysregulation of HPG axis can lead to male subfertility of infertility. GnRH decreases cell proliferation by inhibiting the AKT/ERK1/2 pathway in pancreatic cancer (Suo *et al.*, 2019). GnRH-II antagonist trptorelix-1 (Trp-1) induces autophagosome formation, AKT phosphorylation reduction and c-Jun NH (2) terminal kinase phosphorylation elevation in prostate cancer cells, indicating that GnRH-II antagonists-mediated autophagic degradation may contribute to the treatment of prostate cancer (Kim *et al.*, 2009). Therefore, GnRH-mediated autophagy pathway may hold promise in the treatment of pancreatic or prostate cancer.

Pituitary tumors can disturb the HPG axis, influencing pre-testicular regulation. Dopamine agonists bromocriptine and cabergoline (CAB) have been utilized for the therapies of pituitary prolactinomas and other neuroendocrine tumors. Low-dose CAB is able to induce autophagy but fails to suppress cell growth. However, combination of chloroquine (CQ, autophagy inhibitor) with CAB facilitates the accumulation of p62/caspase-8/LC3-II, indicating blockage of autophagic cycles and involvement of apoptosis. Collectively, combined use of CAB and CQ may increase clinical effectiveness for the treatment of pituitary adenomas (PAs), making it an attractive option in pituitary tumor-induced male infertility (Lin *et al.*, 2017).

Conversely, DEP domain-containing mechanistic target of rapamycin (mTOR)-interacting protein (DEPTOR), as a key regulator of mTOR, inhibits mTOR Complex 1 (mTORC1) and 2 (mTORC2) by decreasing E3 ubiquitin ligase βTrCP1. DEPTOR enhanced autophagic cell death to confer cells sensitivity to CAB in PA (Yao *et al.*, 2019). In line with this, dopamine receptor D5 (DRD5) activation can inhibit tumor growth by autophagic cell death with mTOR pathway (Leng *et al.*, 2017).

In pituitary prolactinoma, FOXP1-induced lncRNA CLRN1-AS1 represses cell proliferation, promotes apoptosis and inhibits autophagy by inactivation of Wnt/β-catenin signaling, thus acting as a tumor suppressor (Wang *et al.*, 2019).

Low-dose (1.25 μM) tetrandrine (Tet) induces autophagy by down-regulation of MAPK/STAT3 in PA; higher-dose (5.0 μM) Tet induces caspase-dependent apoptosis. Meanwhile, autophagy inhibitors enhance Tet-induced caspase activity and apoptotic cell death, indicating that autophagy inhibition enhances the anti-PA effect of Tet (Lyu *et al.*, 2021).

Schaaf–Yang syndrome (SHFYNG), is a neurodevelopmental disorder with neonatal hypotonia, feeding difficulties, hypogonadism, intellectual disability and sleep apnea, caused by pathogenic variants in the paternal copy of MAGEL2. The *Magel2*-null mouse model and fibroblast cell lines from individuals with SHFYNG exhibit the up-regulation of mTOR and the down-regulation of autophagy, which can be rescued by the mTOR inhibitor rapamycin. mTOR may be a potential target for the treatments of SHFYNG-induced hypogonadism (Crutcher *et al.*, 2019).

Male late-onset hypogonadism (LOH), one of the most common diseases, affects male fertility. Testosterone replacement therapy is the main clinical treatment but has obvious side effects. It has been reported that methylpyrimidine-fused tricyclic diterpene analogs 29 could stimulate autophagy by the AMPK/mTOR pathway and furthermore increase testosterone levels and improve sperm qualities with few side effects in androgen deficiency-aging male rats. Therefore, methylpyrimidine-fused tricyclic diterpene analogs may be used as a new type of potential anti-LOH agent by regulating autophagy (Bai *et al.*, 2019).

Non-obstructive azoospermia (NOA), from pre-testicular and testicular factors, is identified in ∼10% of infertile males. Over-expression of hsa_circ_0000116 is presented in patients with NOA. Furthermore, hsa_circ_0000116 may affect male fertility through a hsa_circ_0000116-miR-449-autophagy-related competing endogenous RNA network, suggesting its predictive value in testicular sperm retrieval (Lv *et al.*, 2020).

Cordycepin (COR), an active constituent of the nutrient powerhouse *Cordyceps* *militaris* Linn., ameliorates age-related testicular dysfunction in rats, including sperm motility, progressiveness and average path/straight line velocity. Meanwhile, the expression of spermatogenesis-related protein, SIRT1, and autophagy-related mTORC1 are ameliorated by COR in aged rats, implying the potential therapeutic value of COR in male sexual dysfunction by regulating mTORC1 (KopalLi *et al.*, 2019).

Aflatoxin B1 (AFB1), a potential endocrine disrupter, reduces serum testosterone (T), LH and FSH levels, and the expression of testosterone biosynthesis-related genes. AFB1 induces Leydig cells apoptosis by suppressing AMPK/mTOR-mediated autophagy flux pathway (Chen *et al.*, 2019). KD of *Beclin 1* decreases expression of StAR (a testosterone biosynthesis marker) and testosterone production in Leydig cells. Rapamycin, an autophagy activator, enhances steroidogenesis in primary Leydig cells from aged, but not young, rats (Li *et al.*, 2011). Therefore, autophagic deficiency is related to steroidogenic declines in aged rat Leydig cells.

Excessive activation of autophagy is linked with testicular cell injuries induced by environmental endocrine disruptors. Co-exposure to fluoride and arsenic disrupts the intestinal flora balance and induces testicular autophagy by altering autophagic flux, increasing Beclin1 and LC3-II expression, and decreasing p62 expression in offspring rats (Liu *et al.*, 2021). Perfluoroundecanoic acid (PFUnA), one of long-chain perfluoroalkyl carboxylic acids, downregulates Lhb and Fshb expression in the pituitary and serum FSH/LH/testosterone levels. Meantime, PFUnA reduces Leydig cell number and induces autophagy by suppressing the phosphorylation of mTOR, AKT1/2 and ERK1/2 in the testis of pubertal male rats (Yan *et al.*, 2021).

Apart from the clinical impacts on pre-testicular and testicular diseases, autophagy pathway-related activators and inhibitors may be potential therapies for post-testicular diseases, especially in erectile dysfunction (ED). ED is a problem getting or keeping an erection for satisfactory sexual performance (Najari and Kashanian, 2016). ED can affect libido (sexual interest), orgasm or ejaculation, leading to accumulation of sperm in the epididymis, with a decline of sperm motility.

ED is common in patients with diabetes mellitus. Increasing evidence shows that diabetes mellitus-induced ED (DMED) involves autophagy. Rapamycin, an mTOR inhibitor, can lead to inhibition of AMPK/mTOR and PI3K/AKT/mTOR pathways by mTORC1 (raptor)/p70S6K but not the mTORC2-related pathway. Lin *et al.* (2018) found that rapamycin significantly ameliorated erectile function and nitric oxide/cGMP pathway repression in rats with DMED by inhibiting mTORC1 (raptor)/p70S6K to promote autophagy and inactivate apoptosis. In parallel, Ding *et al.* (2020) reported that Simvastatin alleviated DMED in rats by enhancing AMPK pathway-induced autophagy.

Similarly, glucagon-like peptide-1 (GLP-1) analog liraglutide is reported to improve erectile function by promoting autophagy with different pathways in rats with DMED. Liraglutide reduces oxidative stress and downregulates Ras homolog gene family (RhoA) and Rho-associated protein kinase (ROCK) 2. Therefore, liraglutide exerts protective effects on ED by regulating oxidative stress, autophagy and the RhoA/ROCK pathway, providing preclinical evidence for a potential treatment for DMED (Yuan *et al.*, 2020).

Furthermore, transplantation of human urine-derived stem cells (USCs) ameliorates erectile function and cavernosal endothelial function by promoting autophagy of corpus cavernosal endothelial cells (CCECs) in rats with DMED. *In vitro*, advanced glycation end products (AGEs) were used to mimic the diabetic situation. AGEs-treated CCECs exhibited fewer LC3 puncta formation and lower LC3-II, BECN1 and PCNA expression but increased p62 and cleaved-caspase3 expression, which could be rescued by USCs. More importantly, the repaired erectile function could be abolished by the autophagy inhibitor 3-MA, demonstrating the future clinical perspectives of autophagy-related molecules in ED (Zhang *et al.*, 2019).

miR-301a-3p-enriched exosome significantly recovers erectile function in rats and corpus cavernous smooth muscle cells (CCSMCs) by stimulating autophagy and inhibiting apoptosis. Interestingly, the overexpression of PTEN or TLR4 reverses the therapeutic effects of miR-301a-3p in CCSMCs, indicating that miR-301a-3p modulates PTEN/HIF-1α/TLR4 signaling, autophagy and apoptosis for the treatment of ED. However, the cross-talk of PTEN/HIF-1α/TLR4 signaling and autophagy remains to be further investigated in ED (Liang *et al.*, 2021).

In contrast, icariside II (ICAII) and metformin (MET) can improve erectile function and down-regulate AGEs and receptor of AGEs (RAGE), but ICAII and MET up-regulate the PI3K/AKT/mTOR signaling to reduce the excessive mitochondrial autophagy of corpus cavernosum smooth muscle cells (CCSMCs) (Zhang *et al.*, 2020a). These data suggest that both autophagy defects and autophagy overshoot can lead to ED. Autophagy may be a critical therapeutic target for DMED.

## Conclusions, outstanding questions and future perspectives

Autophagy is a critical lysosomal pathway that maintains cell function and survival through the degradation of cellular components such as organelles and proteins (Czaja *et al.*, 2013). In specific mammalian organ or system, autophagy plays different functions (Table  II). Herein, we first present a comprehensive overview on the regulation of autophagy on the entire fate of the sperm, including spermatogenesis (consisting of spermatocytogenesis, spermatidogenesis and spermiogenesis), sperm maturation and fertilization. We also emphasize, in different sections, the specific issues that deserve attention by researchers in future studies.

During spermatocytogenesis, autophagy acts as a protective mechanism for mitochondrial abnormalities induced by *Trl* gene mutations in PSC; meanwhile, autophagy exerts its bilateral effects on spermatogonium: pro-death and pro-survival under stress. During spermatidogenesis, autophagy and apoptosis acts as partners to promote spermatocyte death; while in haploid round spermatids, autophagy devotes itself to two distinct events: the clearance of CB material and the maintenance of CB homeostasis synchronously. During spermiogenesis, autophagy participates in acrosome biogenesis, flagella assembly and head shaping, and the removal of cytoplasm from elongating spermatids.

After spermatogenesis, through PDLIM1, autophagy not only affects bES to regulate BTB, but also mediates aES to handle cytoskeleton, governing spermatid movement and release during spermiation. A dramatic change of ROS occurs in sperm during capacitation. Proverbially, ROS regulates autophagy by transcriptional and post-transcriptional regulation, including ROS-FOXO3-LC3/BNIP3-autophagy, ROS-NRF2-P62-autophagy, ROS-HIF1-BNIP3/NIX-autophagy and ROS-TIGAR-autophagy (Li *et al.*, 2015). Nonetheless, so far, there is no direct evidence that autophagy participates in the process of capacitation. It may be a good potential target for exploring the molecular mechanisms in capacitation disruption-induced male infertility.

During fertilization, the most striking cellular events are acrosome reaction and PME. Autophagy may be involved in the acrosome reaction through TPCs. When it comes to PME, three popular mechanisms are involved: (i) both autophagy and UPS contribute to PME and MOs after fertilization; (ii) MOs provide membranes for autophagosome formation and launch autophagy in the process of PME; and (iii) autophagy is absent in PME after fertilization in mice. Actually, autophagy may regulate PME before fertilization in mammals.

Definitely, we really appreciate researchers who have contributed significantly to the study of Atgs KD/KO in mammalian testis (Table  I). They have opened the prelude to the regulation of autophagy on sperm fate, and even verified the specific mechanism of certain autophagy molecules on sperm regulation. Early innovative explorations of eminent investigators helped us to prepare this article for reproductive biologists interested in understanding the intriguing regulation of autophagy on the fate of sperm.

Future studies are needed to answer the following questions:

Whether and how do mTORC1 and mTORC2 synergistically orchestrate BTB dynamics by intercellular adhesion and cytoskeleton*?*
How does autophagy exert its bilateral effects, pro-death and pro-survival, in environmental toxicant-induced spermatogonial cell injury*?*
What is the cross-talk between meiosis and autophagy-related proteins during spermatidogenesis*?*
Whether and how does autophagy participate in capacitation*?*
Despite the interaction of TPC2 and mTORC1, whether is there direct evidence that autophagy operates the acrosome reaction through TPCs*?*
What is the interaction mechanism between K63 chains and K48 chains in PME or MOs clearance*?*
How and when is sperm mtDNA eliminated in mammals, and is there direct evidence that autophagy participates in PME or MOs clearance*?*
What is the function of Atgs with no KD/KO model in the testis (Table  I)*?*

Answers to these questions, will be beneficial to interpret the roles of autophagy in the fate of sperm, but may also be helpful to diagnose and treat cases of relevant male infertility.

## Data availability

The data underlying this article are available in the article.
